# Psychiatric disorders and reoffending risk in individuals with community sentences in Sweden: a national cohort study

**DOI:** 10.1016/S2468-2667(22)00312-7

**Published:** 2023-01-17

**Authors:** Denis Yukhnenko, Nigel Blackwood, Paul Lichtenstein, Seena Fazel

**Affiliations:** a Department of Psychiatry, University of Oxford, Oxford, UK; b Institute of Psychiatry, Psychology, and Neuroscience, King's College London, London, UK; c Department of Medical Epidemiology and Biostatistics, Karolinska Institutet, Solna, Sweden

## Abstract

**Background:**

Community sentences are widely used in many countries, often comprising the majority of criminal justice sanctions. Psychiatric disorders are highly prevalent in community-sentenced populations and are thus potential targets for treatment interventions designed to reduce reoffending. We examined the association between psychiatric disorders and reoffending in a national cohort of individuals given community sentences in Sweden, with use of a sibling control design to account for unmeasured familial confounding.

**Methods:**

We did a longitudinal cohort study of 82 415 individuals given community sentences between Nov 1, 1991, and Dec 31, 2013, in Sweden using data from population-based registers. We calculated hazard ratios (HRs) for any reoffending and violent reoffending with Cox regression models. We compared community-sentenced siblings with and without psychiatric disorders to control for potential familial confounding. Additionally, we calculated population attributable fractions to assess the contribution of psychiatric disorders to reoffending behaviours. The primary outcomes of the study were any (general) reoffending and violent reoffending.

**Findings:**

Between Nov 1, 1991, and Dec 31, 2013, those given community sentences who were diagnosed with any psychiatric disorder had an increased reoffending risk in men (adjusted HR 1·59, 95% CI 1·56–1·63 for any reoffending; 1·60, 1·54–1·66 for violent reoffending) and women (1·71, 1·61–1·82 for any reoffending; 2·19, 1·88–2·54 for violent reoffending). Risk estimates remained elevated after adjustment for familial confounding. Schizophrenia spectrum disorders, personality disorders, and substance use disorders had stronger associations with violent reoffending than did other psychiatric disorders. Assuming causality, the adjusted population attributable risk of psychiatric disorders on violent reoffending was 8·3% (95% CI 6·6–10·0) in the first 2 years of community follow-up in men and 30·9% (22·7–39·0) in women.

**Interpretation:**

Psychiatric disorders were associated with an increased risk of any reoffending and violent reoffending in the community-sentenced population. The magnitude of the association between psychiatric disorders and reoffending varied by individual diagnosis. Substance use disorders had the highest absolute and relative risks. Most of the increased risk for any reoffending in individuals with psychiatric disorders could be attributed to comorbid substance misuse. Given their high prevalence, substance use disorders should be the focus of treatment programmes in community-sentenced populations.

**Funding:**

Wellcome Trust.

## Introduction

Community sentences are non-custodial sanctions^1^ and are frequently imposed.^2^ In England and Wales in 2020, for example, only 7% of all sanctions were immediate imprisonment and the remaining 93% comprised community sentences, suspended custodial sentences, and, most frequently, fines.^3^ This community-sentenced population often represents the bulk of the probation service's supervisory workload; in 2020, in England and Wales, this equated to 93 600 individuals compared with 65 458 individuals on post-custodial supervision.^4^ In the USA, this proportion is higher, and in 2019 there were nearly 3·5 million community-sentenced individuals under probation supervision compared with around 900 000 individuals under post-custodial supervision.^5^

Community sentences form a heterogeneous group of sanctions, including probation supervision with and without mandatory treatment requirements for substance misuse and mental health problems. Such sentences are associated with lower reoffending rates than are custodial sentences^1^, ^6^ and reduced economic costs.^7^ Community sentences have been recommended for people with psychiatric disorders.^8^, ^9^, ^10^ Legal grounds for community sentences vary but there are similarities between jurisdictions. Community sentences are typically imposed for less serious offences that do not meet a custodial threshold or for first-time offences. Community-sentenced individuals can maintain employment and family ties, and have access to public education, health care, and welfare systems.

Psychiatric disorders are common in the community-sentenced population.^11^ However, the extent to which these disorders affect reoffending remains uncertain. In general population samples, a two-to-four times increased risk of reoffending in individuals with psychiatric disorders has been reported in systematic reviews.^12^ Previous work has shown that individual psychiatric disorders have varying pathways to violence and persistent criminal behaviour.^13^, ^14^ In people with schizophrenia, distinct pathways have been described that involve interactions between childhood and environmental factors.^15^ Early developmental factors, such as child maltreatment, are difficult to measure directly. However, an indirect way to partly account for such effects and to control for familial confounding is to use sibling designs.^16^

**Research in context**

**Evidence before this study**

We searched PubMed between Jan 1, 1966, to Jan 20, 2022, without language restrictions, for articles on mental health risk factors for criminal recidivism in adults given community sentences. We used the following search terms: “risk AND mental health AND (recidivism OR reoffending OR re-offending) AND (community OR probation).” Our search identified only one systematic review of 15 primary studies. Other screened publications included reviews of intervention studies, primary intervention trials, theoretical papers, observational studies of selected subpopulations of community-sentenced individuals (adolescents, older people, one sex, sexual offenders, or individuals with mental health disorders), or people who have been released from prison. The identified systematic review reported pooled associations between several modifiable (dynamic) risk factors and criminal recidivism in adult community-sentenced populations. The identified modifiable risk factors included mental health needs (odds ratio 1·4, 95% CI 1·2–1·6) and substance misuse (2·3, 1·1–4·9), but these were not based on standard diagnostic categories. Only one study included in the systematic review relied on confirmed medical diagnoses of substance use and other psychiatric disorders. No identified studies considered the potential effect of familial confounding on the association between mental health risk factors and reoffending.

**Added value of this study**

To our knowledge, this is the largest cohort study to examine the association between psychiatric disorders and reoffending in community-sentenced individuals using confirmed medical diagnoses. This is also the largest reoffending study to use a sibling design to partly control for unmeasured early developmental and other familial factors. We provided population estimates for the association between individual psychiatric disorders and reoffending. We found consistently elevated associations with individual psychiatric disorders, which remained after accounting for familial confounding. The effects of psychiatric disorders were typically stronger for violent reoffending than for any (general) reoffending outcomes. Furthermore, this study provides important new evidence on comorbid substance misuse as the strongest modifiable mental health risk factor for reoffending outcomes in community-sentenced individuals.

**Implications of all the available evidence**

Our findings underscore the need for improved detection of psychiatric disorders and wider implementation of accessible mental health and substance use treatments for individuals given community sentences. Further research into the mechanisms of the reported associations and how they can be translated into specialised diversion and rehabilitation programmes is needed.

A systematic review of 15 studies in community-sentenced individuals^17^ reported that modifiable risk factors were associated with criminal recidivism in community-sentenced populations, including mental health needs and substance misuse. The included studies adjusted for measured sociodemographic confounders but did not consider familial confounding. This omission is an evidence gap because reported associations might not be secondary to mental ill health or substance misuse, but instead to other co-occurring factors, such as parental criminality or socioeconomic background, which themselves are linked with criminal behaviour in offspring.^18^

To address previous evidence gaps, we did a population-based longitudinal study of individuals given community sentences. We used a sibling control design to account for familial confounding. We addressed three questions. First, whether being diagnosed with a psychiatric disorder was independently associated with any (general) and violent reoffending in community-sentenced individuals. Second, whether this association differed by psychiatric diagnosis. Third, the extent to which any observed associations were explained by comorbid substance use disorder.

## Methods

### Study design and participants

In this cohort study, we followed STROBE guidelines^19^ for the reporting of observational studies (appendix pp 1–2). We linked the following longitudinal, nationwide Swedish registers: the National Crime Register, containing information about criminal offences and convictions since 1973; the National Patient Register, providing information about diagnoses for individuals admitted to inpatient hospitals (since 1973) and outpatient care (since 2001); the Migration Register, containing dates of migration to and from Sweden; the Cause of Death Register, containing information about deaths and causes of deaths (since 1958); the Multi-Generation Register, containing information about biological relationships for all individuals living in Sweden (since 1933); and the Longitudinal Integrated Database for Health Insurance and Labour Market Studies, containing yearly estimations of income, marital and employment status, and education (since 1990). In Sweden, all residents (including immigrants) have a unique personal identifier used in national registers, enabling data linkage.^20^ Data about community sentences in the National Crime Register were sourced from the Swedish Prison and Probation Service. Further information on probation is given in the appendix (p 3).

We included all adult (18 years or older) Swedish residents who received any community sentence at any point from Nov 1, 1991, to Dec 31, 2013. We chose this starting point to ensure full availability of sociodemographic information for all selected individuals, as this information was only available in registers commencing in 1990. Community sentences included conditional sentences with community service, probation with community service, and probation with contracted treatment. We only selected individuals whose sentences came into legal force and were not appealed or dismissed. For each individual, we used the date when a community sentence came into force as the start of follow-up. If an individual had multiple community sentences recorded in the system, we randomly selected one of the sentences to follow up. Exclusion criteria are listed in the appendix (p 4). We additionally identified same-sex full siblings within the cohort using the Multi-Generation Register. The flowchart for this selection process is included in the appendix (p 5). The study was approved by the Regional Ethics Committee at the Karolinska Institutet (2013/5:8). Written consent from participants was not required as the study was conducted on anonymised routinely collected population register data and received ethics approval on this basis.

### Measures

We extracted sociodemographic, criminal, and medical history information at the start of the community sentence. The sociodemographic information included sex, age, education, marital status, employment, and income support.

Criminal history included the dates of all previous convictions and their corresponding crime codes. We separately recorded if the index sentence was an individual's first conviction or if they were previously sentenced, and the previous and index (current) violent offence. A violent offence was defined as a homicide, assault, robbery, arson, any sexual offence (rape, sexual coercion, child molestation, indecent exposure, or sexual harassment), illegal threat, or intimidation.

Medical history included any psychiatric diagnosis received before the index sentence. In line with previous research,^21^ we used a hierarchical approach to diagnostic categories, as follows: schizophrenia spectrum disorders, bipolar disorder, depression, and anxiety disorder. If an individual had a diagnosis of schizophrenia and other diagnoses, they were classified as having schizophrenia. If an individual did not have schizophrenia but had bipolar disorder and depression or anxiety, they were classified as having bipolar disorder. Therefore, each individual could only be classified as having one of the four diagnoses. This approach enables the exclusion of disorders whose symptoms can be subsumed or caused by another disorder.^22^

To explore the effects of comorbidity between psychiatric disorders,^23^ we also investigated alcohol use disorder, drug use disorder, personality disorder, attention-deficit hyperactivity disorder, and other developmental or childhood disorders. We did not use a hierarchical approach for these comorbidities but examined whether they were present or not. International Classification of Diseases diagnostic codes are listed in the appendix (p 6). To assess the cumulative effect of multiple diagnoses, we recorded the number of distinct diagnostic categories per person. We additionally coded the substance use disorder category as having either alcohol use disorder, drug use disorder, or both. To further examine the association between substance use disorder comorbidity and reoffending, we compared individuals who had a psychiatric diagnosis with and without comorbid substance use disorder to control individuals without any psychiatric diagnosis.

Of 82415 individuals in the cohort, 578 (0·7%) had no demographic data and 3335 (4·0%) did not have education data at baseline. We did not replace missing data by imputation or other methods because the amount of missing data was small. In a sensitivity analysis, we recalculated the results with missing values imputed ten times using the Amelia package for R.^24^ Estimated coefficients were combined across imputations using Rubin's rule.^25^ Other sensitivity analyses examined time periods and alternative sibship definitions (appendix pp 7–19).

### Outcomes and censoring

The primary outcomes were any (general) reoffending and violent reoffending. We defined general reoffending as committing any offence after the index sentence date until Dec 31, 2013. We defined violent reoffending as committing a violent offence (a homicide, assault, robbery, arson, any sexual offence, illegal threat, or intimidation) within the same period. Dates of crimes are recorded in registers retroactively after the circumstances of the crime have been established by a court. If no date for the offence was recorded, we used the court sentence date. All individuals were followed up until their first new offence, death, permanent emigration from Sweden, or the end of follow-up.

### Statistical analysis

To examine the association between psychiatric disorders and the risks of general and violent reoffending, we compared community-sentenced individuals with and without psychiatric disorders. We used the Cox proportional hazard model as the method of quantifying this association and Kaplan-Meier survival curves. We tested proportional hazards assumptions by visually examining the Kaplan-Meier curves and Schoenfeld residuals diagrams. The plots were created in R using the survminer package.^26^ If the date of a new offence was the same as the date of the start of the index sentence, we changed the end time of 0 to the end time of 0·5 days.^27^ The analyses were completed in R using the survival package.^28^ The data structure, formula, and code for sibling analysis are presented in the appendix (p 20).

All analyses were stratified by sex because of theoretical and practical considerations. Theoretical considerations constitute potentially different pathways for violent and criminal behaviour in men and women.^29^ Additionally, men and women are often dealt with separately in the criminal justice system, and the trend towards gender-informed services might benefit from this information.

We estimated the association between psychiatric disorders and reoffending by fitting two Cox regression models. In the first model, we adjusted only for age at the time of the sentence. We chose models adjusted by age and stratified by sex as the primary model to estimate the total effect of given exposures (psychiatric disorders) and outcomes (general and violent reoffending). Given the possible bidirectional relationship between many of our measured covariates, the inclusion of a large number of the covariates in the model could lead to overadjustment, with the resulting estimates likely being biased and hard to interpret.^30^ In the first model, which was fitted on the full cohort, we did not perform any familial clustering or stratification.

In the second model, we fitted a fixed-effect Cox regression model^31^ to the subsamples of same-sex full siblings receiving a community sentence but discordant for a given psychiatric diagnosis. Therefore, for a sibship to be included in the sibling analysis cohort, at least two siblings from one family had to be given community sentences at some point in their life. The fixed-effect model adjusted for all unmeasured genetic and environmental factors that were shared between siblings, provided their effect remained constant over time. Previous research has shown that adverse childhood experiences, such as parental neglect, sexual abuse, and (to a lesser extent) physical abuse, are highly correlated among siblings.^32^, ^33^ Comparison of discordant siblings allowed us to control for confounding arising from stable familial factors shared between siblings.^34^ To further assess the effect of psychiatric disorders on criminal reoffending behaviours, we calculated the population attributable fraction (PAF). The PAF estimates the proportion of new offences that can be attributed to a risk factor, with the assumption that a causal association exists. To calculate the PAF, we used the model-based adjusted attributable fraction function for Cox proportional hazard models in the AF package in R.^35^ We additionally estimated PAF adjusting for measured criminal history and sociodemographic factors. We restricted the estimation of PAF to the first 2 years of the follow-up period and fitted the models separately for men and women. The data structure and code for PAF calculations are presented in the appendix (p 21).

We compared individuals diagnosed with mental disorders with and without comorbid substance misuse to individuals without a psychiatric history by fitting Cox regression models (ie, we selected the individuals with a given diagnosis and comorbid substance misuse and compared reoffending risk to those without a psychiatric diagnosis). Additionally, we selected individuals with a given diagnosis without comorbid substance misuse and compared reoffending risk to those without a psychiatric diagnosis. We also estimated the effect of having multiple psychiatric diagnoses on reoffending in individuals with and without a substance use disorder diagnosis.

All analyses were done in R Studio version 1.4.1106 using R 4.0.5.

### Role of the funding source

The funders of the study had no role in study design, data collection, data analysis, data interpretation, or writing of the report.

## Results

Between Nov 1, 1991, and Dec 31, 2013, 82 415 individuals (70 643 [85·7%] men and 11 772 [14·3%] women) received at least one community sentence in Sweden. For general reoffending, the median follow-up was 23 months (IQR 7·6–54·9). For violent reoffending, the median follow-up was 39 months (15·1–73·6). 33 774 (47·8%) of 70 643 men committed a new offence during follow-up, and 10 591 (15·0%) committed a new violent offence. 4334 (37·8%) of 11 772 women committed a new offence (general reoffending) and 868 (7·4%) committed a new violent offence during follow-up (see appendix p 22 for survival estimates).

At baseline, 7062 (60·0%) of 11 772 women had been diagnosed with any psychiatric disorder compared with 27 138 (38·4%) of 70 643 men. Baseline characteristics are presented in table 1 and univariate analyses for general reoffending and violent reoffending are shown in the appendix (pp 23–26). Psychiatric comorbidity was common (appendix p 27). The same-sex full sibling cohort and differences with the primary analysis cohort are presented in the appendix (pp 28–29).

**Table 1:** Baseline characteristics and follow-up data of adults receiving community sentences from Nov 1, 1991, to Dec 31, 2013

		**Men (n=70 643)**	**Women (n=11 722)**	**Total (n=82 415)**
**Baseline**				
Age, years		27 (22–38)	31 (23–41)	28 (22–38)
Married or in a registered partnership		7453 (10·6%)	1642 (14·0%)	9095 (11·0%)
Employed		31 153 (44·1%)	3909 (33·3%)	35 062 (42·5%)
Years of education				
	<9	2322 (3·3%)	500 (4·3%)	2822 (3·4%)
	9–11	60 649 (85·9%)	9705 (82·8%)	70 354 (85·4%)
	≥12	4760 (6·7%)	1144 (9·8%)	5904 (7·2%)
Recipient of income support		24 367 (34·5%)	5634 (48·1%)	30 001 (36·4%)
Previous criminal history		54 395 (76·9%)	7832 (66·8%)	62 227 (75·5%)
Previous violent crime		27 222 (38·5%)	2373 (20·2%)	29 595 (35·9%)
Previous imprisonment		15 755 (22·3%)	1392 (11·9%)	17 147 (20·8%)
Index violent offence		32 941 (46·6%)	4013 (34·2%)	36 954 (44·8%)
Any psychiatric disorder		27 138 (38·4%)	7062 (60·2%)	34 200 (41·5%)
Any psychiatric disorder (excluding substance use)		18 047 (25·5%)	5486 (46·8%)	23 533 (28·6%)
	Schizophrenia spectrum disorder	2032 (2·9%)	563 (4·8%)	2595 (3·1%)
	Bipolar disorder	690 (1·0%)	340 (2·9%)	1030 (1·2%)
	Depression	5447 (7·7%)	2037 (17·4%)	7484 (9·1%)
	Anxiety disorder	5604 (7·9%)	1869 (15·9%)	7473 (9·1%)
	Personality disorder	2671 (3·8%)	1324 (11·3%)	3995 (4·8%)
	Attention-deficit hyperactivity disorder	3370 (4·8%)	608 (5·2%)	3978 (4·8%)
	Other developmental or childhood disorder	3246 (4·6%)	777 (6·6%)	4023 (4·9%)
Substance (drug or alcohol) use disorder		18 680 (26·4%)	4825 (41·2%)	23 505 (28·5%)
	Alcohol use disorder	11 569 (16·4%)	2961 (25·3%)	14 530 (17·6%)
	Drug use disorder	11 864 (16·8%)	3345 (28·5%)	15 209 (18·5%)
**Follow-up data: general reoffending**				
Number of person-years at risk		201 415·6	38 800·4	240 216·0
Incidents of general reoffending during follow-up		33 774 (47·8%)	4434 (37·8%)	38 208 (46·4%)
Time to any new offence, months				
	All individuals	22·4 (7·4–53·0)	28·2 (9·7–61·6)	23·2 (7·6–54·9)
	Individuals with psychiatric disorder	15·7 (5·1–39·8)	22·8 (6·8–52·2)	17·1 (5·4–43·2)
	Individuals without psychiatric disorder	27·4 (9·4–60·5)	38·2 (13·5–74·0)	28·2 (9·7–61·6)
General reoffending rate (cumulative)				
	1-year	18 019 (25·5%)	2427 (20·6%)	20 446 (24·8%)
	2-year	24 654 (34·9%)	3234 (27·6%)	27 888 (33·8%)
	3-year	27 992 (39·6%)	3666 (31·3%)	31 658 (38·4%)
	4-year	30 012 (42·5%)	3927 (33·5%)	33 939 (41·2%)
	5-year	31 351 (44·4%)	4104 (35·0%)	35 455 (43·0%)
Died during follow-up		1024 (1·4%)	179 (1·5%)	1203 (1·5%)
Emigrated during follow-up		750 (1·1%)	106 (0·9%)	856 (1·0%)
**Follow-up data: violent reoffending**				
Number of person-years at risk		276 437·0	53 244·5	329 681·5
Incidents of violent reoffending during follow-up		10 591 (15·0%)	868 (7·4%)	11 459 (13·9%)
Median time to a violent offence (months)				
	All individuals	37·7 (14·5–72·4)	46·5 (20·3–82·9)	38·7 (15·1–73·6)
	Individuals with psychiatric disorder	29·9 (10·8–61·4)	41·9 (16·6–75·7)	32·1 (11·8–64·1)
	Individuals without psychiatric disorder	41·9 (16·6–75·7)	55·6 (25·5–90·3)	44·8 (18·8–80·0)
Violent reoffending rate (cumulative)				
	1-year	4030 (5·7%)	300 (2·6%)	4330 (5·3%)
	2-year	6219 (8·8%)	467 (4·0%)	6686 (8·1%)
	3-year	7552 (10·7%)	572 (4·9%)	8124 (9·9%)
	4-year	8462 (12·0%)	657 (5·6%)	9119 (11·1%)
	5-year	9077 (12·8%)	711 (6·1%)	9788 (11·9%)
Imprisoned during follow-up		9802 (13·9%)	1082 (9·2%)	10 884 (13·2%)
Died during follow-up		1655 (2·3%)	324 (2·8%)	1979 (2·4%)
Emigrated during follow-up		1027 (1·5%)	152 (1·3%)	1179 (1·4%)

Data are median (IQR), n (%), or n. 510 men and 58 women have missing values for marital status, employment, and income support. 2912 men and 423 women have missing values for education.

**Figure 1:**
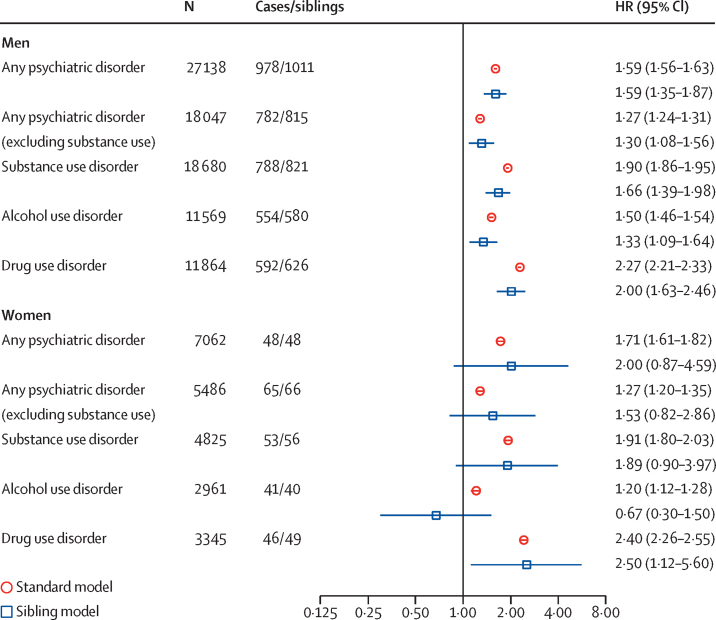
Association between psychiatric disorders and general reoffending in individuals given community sentences, stratified by sex HRs in the standard model were adjusted for age. The sibling model is a fixed-effect model adjusted for age and any unmeasured covariates shared between siblings discordant by a given risk factor. Cases indicates the number of sibling probands with a given diagnosis. Siblings indicates the number of siblings, discordant by a given diagnosis with their proband. HR=hazard ratio.

Having any previous psychiatric diagnosis at the start of a community sentence was associated with an increased risk of general reoffending (figure 1; appendix pp 30–31). Individual psychiatric diagnoses were typically associated with an increased risk of general reoffending in both men and women (appendix p 31).

In men, hazard ratios [HRs] for individual disorders ranged from 1·02 to 2·27, and 24 654 (34·9%) of 70 643 individuals reoffended for any crime during the first 2 years of the follow-up period, of which 3349 new offences were potentially attributable to psychiatric disorders. Assuming causality, this corresponds to a PAF of 5·5% (95% CI 4·8–6·3), adjusted for age, criminal history, and sociodemographic factors (appendix p 32). Sibling analyses showed that men with schizophrenia spectrum disorder, anxiety disorder, alcohol use disorder, and drug use disorder had a significantly higher risk of general reoffending compared with their same-sex full siblings, discordant for the individual diagnosis investigated (figure 1; appendix p 31).

In women, HRs for individual disorders ranged from 0·87 to 2·40, and 3234 (27·5%) of 11 772 individuals reoffended for any crime during the first 2 years of the follow-up period, of which 822 new offences were potentially attributable to psychiatric disorders. Assuming causality, this corresponds to an adjusted PAF of 15·7% (95% CI 12·5–18·9; appendix p 32).

The female cohort contained a relatively small number of same-sex full siblings discordant by a given diagnosis. Thus psychiatric associations had wide CIs that crossed 1, with the exception of drug use disorder (figure 1).

Among people with psychiatric diagnoses, substance misuse comorbidity was associated with a higher risk of general reoffending compared with individuals without substance misuse comorbidity (table 2). However, individual disorders without substance misuse comorbidity were mostly not significantly associated with general reoffending.

**Table 2:** General reoffending in individuals given community sentences with a psychiatric disorder with and without substance misuse comorbidity

	**Incidence of general reoffending**		HR (95% CI)*	
	With substance use	Without substance use	With substance use	Without substance use
**Men (n=70 643)**				
Any psychiatric disorder	10 777/18 680 (57·8%)	3504/8458 (41·4%)	1·92 (1·87–1·96)	1·07 (1·03–1·11)
Schizophrenia spectrum	791/1368 (57·8%)	248/664 (37·3%)	2·29 (2·13–2·46)	0·95 (0·84–1·08)
Bipolar	181/451 (40·1%)	85/239 (35·6%)	1·53 (1·32–1·77)	1·01 (0·82–1·25)
Depression	1534/3220 (47·6%)	774/2227 (34·8%)	1·61 (1·53–1·70)	0·92 (0·85–0·98)
Anxiety	1573/2841 (55·4%)	1126/2763 (40·8%)	2·03 (1·93–2·14)	1·07 (1·00–1·13)
**Women (n=11 772)**				
Any psychiatric disorder	2266/4825 (47·0%)	720/2237 (32·2%)	2·01 (1·88–2·14)	1·18 (1·08–1·29)
Schizophrenia spectrum	192/369 (52·0%)	55/194 (28·4%)	2·51 (2·15–2·93)	1·03 (0·79–1·35)
Bipolar	75/234 (32·1%)	25/106 (23·6%)	1·52 (1·20–1·92)	0·86 (0·58–1·28)
Depression	481/1255 (30·38%)	232/782 (30·0%)	1·69 (1·52–1·87)	1·11 (0·97–1·28)
Anxiety	476/1050 (45·3%)	269/819 (32·8%)	1·96 (1·76–2·17)	1·20 (1·05–1·37)

Data are number of individuals reoffending/number of individuals with disorder (%), unless otherwise indicated. HRs are adjusted for age; individuals without psychiatric disorders were used as the reference. HR=hazard ratio.

* Cox regression models were fitted separately in individuals who had a given psychiatric disorder with comorbid substance misuse and individuals who had a given psychiatric disorder without comorbid substance misuse; in both cases, individuals without substance use or other psychiatric disorders were a reference group.

In men and women, being diagnosed with multiple psychiatric disorders (other than drug or alcohol use disorders) was associated with an increased risk of general reoffending (appendix p 33). The risk increased in a stepwise manner with each additional diagnosis. However, when individuals with and without comorbid substance misuse were analysed separately, the stepwise increase in the risk of general reoffending was no longer present.

Having any previous psychiatric diagnosis at the start of a community sentence was associated with an increased risk of violent reoffending (appendix pp 34–35). Individual psychiatric diagnoses were typically associated with an increased risk of violent reoffending in men and women (appendix p 35).

In men, HRs for individual disorders ranged from 1·13 to 2·18 (appendix p 35). 6219 (8·8%) men violently reoffended during the first 2 years of the follow-up, and 1039 violent reoffences were attributable to psychiatric disorders (table 1). Assuming causality, this finding corresponds to an adjusted PAF of 8·3% (95% CI 6·6–10·0; appendix p 32).

**Figure 2:**
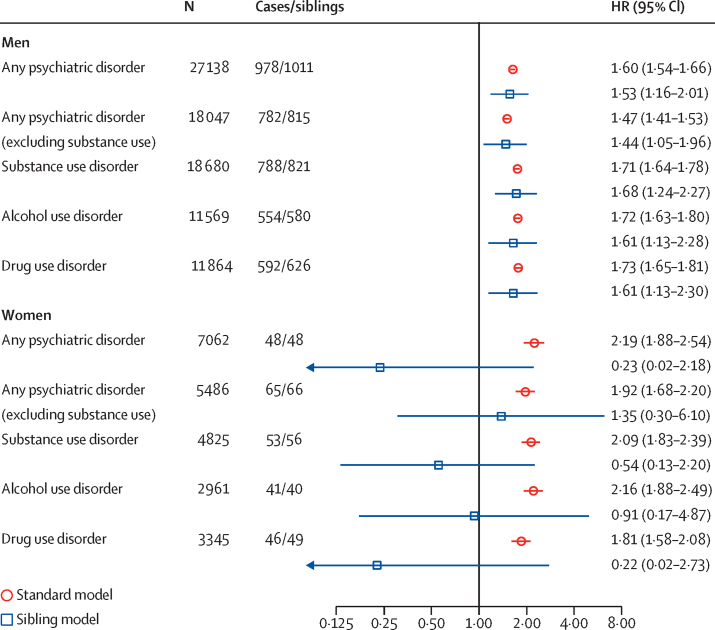
Association between psychiatric disorders and violent reoffending in individuals given community sentences, stratified by sex HRs in the standard model were adjusted for age. The sibling model is a fixed-effect model adjusted for age and for any unmeasured covariates shared between siblings discordant by a given risk factor. Cases indicates the number of sibling probands with a given diagnosis. Siblings indicates the number of siblings, discordant by a given diagnosis with their proband. HR=hazard ratio.

Sibling analyses showed that men with schizophrenia spectrum disorder, personality disorder, alcohol use disorder, and drug use disorder had a higher risk of violent reoffending compared with their same-sex full siblings (figure 2; appendix p 35).

In women, all individual psychiatric diagnoses, except for bipolar disorder and depression, were associated with an increased risk of violent reoffending. HRs for individual disorders in women ranged from 1·09 to 2·61 (appendix p 35). Overall, in the female cohort, 457 (4·0%) individuals violently reoffended during the first 2 years of the follow-up, and 188 new violent offences were potentially attributable to psychiatric disorders (table 1). Assuming causality, this finding corresponds to an adjusted PAF of 30·9% (95% CI 22·7–39·0; appendix p 32). For the female cohort, there were a small number of same-sex full siblings, discordant by a given diagnosis, which meant that reported associations had wide CIs.

Among men and women with psychiatric disorders, individuals with substance misuse comorbidity had a higher risk of violent reoffending compared with individuals without such comorbidity (table 3). In men and women, having multiple psychiatric diagnoses (other than drug or alcohol use disorders) was associated with an increased risk of violent reoffending (appendix p 33), which increased in a stepwise manner per additional diagnosis, with and without comorbid substance misuse.

**Table 3:** Violent reoffending in individuals given community sentences with a psychiatric disorder with and without substance misuse comorbidity

	**Incidence of violent reoffending**		HR (95% CI)*	
	With substance use	Without substance use	With substance use	Without substance use
**Men (n=70 643)**				
Any psychiatric disorder	3272/18 680 (17·5%)	1264/8458 (14·9%)	1·78 (1·70–1·86)	1·30 (1·22–1·38)
Schizophrenia spectrum	314/1368 (23·0%)	111/664 (16·7%)	2·83 (2·52–3·17)	1·53 (1·27–1·85)
Bipolar	50/451 (11·1%)	32/239 (13·4%)	1·56 (1·18–2·06)	1·37 (0·96–1·93)
Depression	476/3220 (14·8%)	244/2227 (11·0%)	1·69 (1·54–1·86)	1·01 (0·89–1·15)
Anxiety	490/2841 (17·2%%)	385/2763 (13·9%)	1·99 (1·81–2·19)	1·24 (1·12–1·37)
**Women (n=11 772)**				
Any psychiatric disorder	479/4825 (9·9%)	153/2237 (6·8%)	2·48 (2·12–2·90)	1·65 (1·35–2·03)
Schizophrenia spectrum	61/369 (16·5%)	21/194 (10·8%)	5·01 (3·75–6·71)	3·26 (2·06–5·17)
Bipolar	18/234 (7·7%)	6/106 (5·7%)	2·94 (1·81–4·79)	1·53 (0·68–3·45)
Depression	97/1255 (7·7%)	41/782 (5·2%)	2·27 (1·79–2·89)	1·38 (0·99–1·93)
Anxiety	117/1050 (11·1%)	61/819 (7·4%)	2·97 (2·37–3·71)	1·82 (1·37–2·41)

Data are number of individuals reoffending/number of individuals with disorder (%), unless otherwise indicated. HRs are adjusted for age; individuals without psychiatric disorders were used as the reference. HR=hazard ratio.

* Cox regression models were fitted separately in individuals who had a given psychiatric disorder with comorbid substance misuse and individuals who had a given psychiatric disorder without comorbid substance misuse. In both cases, the individuals without substance use or other psychiatric disorders were a reference group.

There were no differences when the data on sociodemographic characteristics were imputed. Some differences in the strength but not direction of associations with psychiatric disorders by period were found (index community sentence during 1991–2001 *vs* 2001–05 *vs* 2006–13; appendix pp 9–16). Sibling analysis of discordant pairs showed similar results to full sibships (appendix pp 7–8).

## Discussion

In this cohort study, we examined the association between psychiatric disorders and reoffending in a Swedish nationwide population-based study of 82 386 individuals given community sentences over 14 years. We followed up individuals from the day of their community sentence until the date of a new offence and examined the reoffending risk in men and women. Our study had four principal findings.

First, psychiatric disorders were significantly associated with an increased risk of general and violent reoffending. These associations could be due to disorder-specific (eg, psychotic symptoms, high impulsivity, and low empathy) and non-specific mechanisms (eg, reduced employment and social support). Comparisons between same-sex siblings discordant by their diagnosis, who were both given community sentences, found little evidence of familial confounding on the association between most psychiatric disorders and reoffending outcomes in men. This finding is consistent with an independent association of individual psychiatric disorders with reoffending, since sibling comparisons account for shared childhood environment and half of their co-segregating genes.^36^ Substantial attenuation of effects in sibling models would suggest that certain psychiatric disorders have common familial causes with reoffending or that the effect of psychiatric disorders is mediated through familial factors,^37^ but we did not observe this. In women, although adjustment for familial confounding led to mostly non-significant risk estimates for individual disorders, low statistical power could explain this finding.

Second, although relative risks differed by psychiatric diagnosis, substance use disorder was the diagnostic category associated with the highest absolute risks. In the study cohort, 23 505 (28·5%) of 82 415 individuals were previously diagnosed with drug or alcohol use disorder. Furthermore, during follow-up, individuals with any psychiatric diagnosis (including substance use disorder) had 5168 cases of violent reoffending, 3751 (73%) of which were committed by individuals with substance use disorder. For some crimes, this finding is unsurprising, as substance misuse might be directly related to drug possession or substance intoxication, but the associations with violent crime are noteworthy.

Third, the magnitude of the association between psychiatric disorders and reoffending varied by individual diagnosis. Schizophrenia spectrum disorders, personality disorders, and substance use disorders were more strongly associated with violent offending than were other disorders. Schizophrenia spectrum disorders were associated with both general and violent reoffending, which remained significant after adjustment for familial confounding. However, these findings were not present for mood disorders. Our findings differed from those reported for released prisoners,^21^ but were consistent with previous research in community-sentenced individuals.^38^ A potential explanation is that different symptoms contribute to criminality in lower-risk versus higher-risk individuals with mood disorders. For example, in individuals with bipolar disorder, a predominantly manic illness course with antisocial and impulsive traits increases the risk of offending.^39^ Thus, such individuals might be more likely to commit severe violent offences leading to imprisonment rather than community sentences.

Fourth, most of the increased risk for general reoffending in individuals with mental health disorders could be attributed to comorbid substance misuse. However, in violent reoffending, such comorbidity only partly explained the association, which has also been reported in people who have been released from prison.^21^ In some disorders, such as schizophrenia or related psychoses, certain psychotic symptoms, for example, persecutory delusions, might be specifically associated with violence but not general criminality. Relationships between substance misuse, psychiatric disorders, and reoffending are complex and will vary between individuals.^40^ In some individuals, substance misuse can trigger or cause psychiatric symptoms.^41^ In others, psychiatric symptoms might predate substance misuse problems and increase the probability of reoffending.^42^ Substances might exacerbate underlying psychiatric symptoms, such as paranoid ideas, which are linked to violence, and other more general factors, such as disinhibition and hostility, which increase risk. Finally, sourcing substances might lead individuals to commit acquisitive crimes to fund their purchase, and participation in social networks where criminal behaviour is common.^43^

Our study underscores the prominent role of substance use disorders as primary or comorbid conditions in individuals with repeat serious offending. Our results corroborate findings in people who have been released from prison^21^ and in the community.^12^ As substance misuse is highly prevalent in community-sentenced populations,^44^, ^45^ interventions that target drug and alcohol use (rather than general mental health) could lead to a larger absolute reduction in recidivism than for other disorders. Evidence exists for the effectiveness of opioid substitution therapy in community-sentenced individuals.^46^ There is also a small amount of trial support for therapeutic community intervention, but not for other psychosocial treatments.^47^ As a community sentence is often an individual's first point of contact with the criminal justice system, community supervision represents an opportunity for early intervention. We estimate, based on PAFs, that successful substance treatment could have led to a reduction in violent reoffending rates of up to 8% in men and 25% in women. PAFs assume causality, overestimate effects, and should be interpreted with caution, but can indicate the potential public health impact of health-care services. We have examined risk factors using a large nationwide cohort with validated exposures and outcomes with sufficient power to examine individual diagnoses in the primary models for men and women, and in men, using sibling controls.

Several limitations of our study should be noted. We relied on patient registers for ascertainment of psychiatric diagnoses, and did not have outpatient data until 2001. Hence, our approach might lead to a conservative estimate for the prevalence of psychiatric disorders, as more severe cases would be identified in the patient register. In our cohort, individuals with more chronic and enduring diagnoses, such as schizophrenia or bipolar disorder, were less selected as around 90% will access specialist mental health services during a 10-year period,^48^ in contrast to individuals with depression or substance use disorders, for whom secondary care registers will probably capture more severe cases, which might lead to overestimation of the effect of these disorders. Moreover, in sensitivity analyses, we found that the strength of association between psychiatric disorders and reoffending was dependent on time period of sentencing, with stronger associations during 2006–13 compared with previous periods, although this finding was explained by drug misuse. This time period corresponded with a substantial increase in the number and proportion of sentences for drug-related offences handed down in Sweden, together with a change in how diagnoses were ascertained (ie, including outpatient data in the National Patient Register from 2001). Taken together, these changes meant, for individuals with drug use disorder, the probability of being convicted for a drug-related crime, such as purchase or possession, increased from 1991 to 2013. Thus, this particular finding suggests that estimates from the total cohort might, in contrast to the limitations associated with ascertaining diagnoses from registers, be conservative with regard to the contribution of substance misuse.

Because of the observational design of the study, we could not fully account for unobserved confounding of the relationship between substance use disorder and other psychiatric disorders. Findings on comorbidity could be also partly explained by the possibility that substance misuse is a marker for more severe primary mental ill health. Future research using a primary care database could test these effects.

Our research was done in a single country with a freely accessible public health system, which could lead to conservative estimates of the effect of psychiatric disorders on recidivism, as sentenced individuals might receive a higher number of community interventions than in other countries. Community sentencing and recidivism are sensitive to definition, crime detection, and legal practices.^1^ Some generalisability is suggested by the proportion of people sentenced to community probation who have index violent offences in some other countries—for example, 22% in the USA and 23% in Sweden.^5^, ^49^ Another limitation of the study is the absence of contemporaneous data and whether the use of such sentences has changed over time. However, available evidence from Sweden does not suggest major changes. The mean annual number of community sentences has remained similar—11 995 during 1993–2013 and 11 241 during 2014–21. Furthermore, the proportion of all sentences that were community-based was 46% during the study period, which increased marginally to 50% during 2014–21.^50^

Limitations from using sibling comparisons include that the subcohort of full siblings differed in age and offending from the primary analysis cohort. However, the prevalence of psychiatric disorders was similar. Second, sibling comparison methods adjust for shared familial factors only, and siblings can experience different early environments, so the association between exposure and the outcome should be interpreted accordingly. Third, the siblings within the study cohort could potentially show more collinearity in measured covariates than unrelated individuals. However, given that siblings constituted 6% of the cohort and had a similar prevalence of measured exposures, any meaningful effect of within-family dependence on the results would likely be negligible.

Future research could explore links with historical factors, including childhood abuse and past suicidal behaviours,^51^, ^52^ which might help to further explain pathways and mechanisms to offending in adolescence and early adulthood. Targeting the underlying causes of repeat offending can reduce risk.^53^ Given the economic impact of violent crime perpetrated by individuals with severe mental health problems and comorbid substance misuse,^54^ interventions through mental health services will be a cost-effective option. Criminal justice services should consider expanding community-based mental health and substance misuse treatments to reduce reoffending. Such programmes are underutilised in many countries, including England and Wales,^55^ and further integration of probation with mental health services should be prioritised.^56^, ^57^

In conclusion, we found associations between major psychiatric disorders and reoffending outcomes in community-sentenced individuals. Associations remained independent after adjustment for familial factors, and were strongest for primary and comorbid substance use disorders. Our findings suggest the need for wider implementation of accessible and evidence-based treatment programmes for individuals who are given community sentences. Given the high prevalence of substance use and other psychiatric disorders in community-sentenced individuals, such intervention programmes are likely to prevent further criminalisation and improve quality of life.

## Data sharing

The study was done using data from the Swedish population registers. The Public Access to Information and Secrecy Act in Sweden prohibits us from making individual-level data publicly available. Researchers interested in replicating our work can apply for individual-level data from Statistics Sweden (mikrodata@scb.se) for data from The Total Population Register (https://www.scb.se/vara-tjanster/bestallamikrodata/vilka-mikrodata-finns/individregister/registret-over-totalbefolkningen-rtb/), The Multi-Generation Register (https://www.scb.se/varatjanster/bestalla-mikrodata/vilka-mikrodata-finns/individregister/flergenerationsregistret/), and The Longitudinal Integrated Database for Health Insurance and Labour Market Studies (https://www.scb.se/en/services/guidance-for-researchersand-universities/vilka-mikrodata-finns/longitudinella-register/longitudinal-integrateddatabase-for-health-insurance-and-labour-marketstudies-lisa/); The National Board of Health and Welfare (registerservice@socialstyrelsen.se) for data from The National Patient Register (https://www.socialstyrelsen.se/patientregistret); and The Swedish National Council for Crime Prevention (statistik@bra.se) for data from The National Crime Register (https://www.bra.se/statistik/kriminalstatistik/specialbestallningar.html).

## Declaration of interests

We declare no competing interests.
