# Supplementary material for: Psychiatric disorders and reoffending risk in individuals with community sentences in Sweden: a national cohort study
Source: Lancet Public Health. 2023 Jan 17;8(2):e119–29. doi: 10.1016/S2468-2667(22)00312-7 (PMC10914666; doi:10.1016/S2468-2667(22)00312-7)
Supplement: Supplementary appendix [file mmc1.pdf]

THE LANCET
Public Health

Supplementary appendix

This appendix formed part of the original submission and has been peer reviewed. We post it as supplied by the authors.

Supplement to: Yukhnenko D, Blackwood N, Lichtenstein P, Fazel S. Psychiatric disorders and reoffending risk in individuals with community sentences in Sweden: a national cohort study. *Lancet Public Health* 2023; published online Jan 17. https://doi.org/10.1016/S2468-2667(22)00312-7.

Supplementary material

Table of contents

STROBE reporting checklist 1
Additional information about probation in Sweden 3
Additional notes on methods 4
Selection process for the analysis cohort 5
ICD codes for medical history covariates at baseline 6
Results of sibling comparison using sibling pairs discordant by diagnosis 7
Additional sensitivity analyses 9
The analysis for the sub-cohort of individuals sentenced in the period from 2006 to 2013 17
Formula and R code for sibling comparison 20
R code for population attributable fractions (PAF) 21
Kaplan-Meier curves for obversed general and violent reoffending in the full cohort 22
Univariate analysis 23
Collinearity estimates 27
Same-sex full sibling cohort 28
Kaplan-Meier curves for observed general reoffending in individuals given community sentences stratified by sex and psychiatric disorder status 30
Association between individual psychiatric diagnoses and general reoffending in individuals given community sentences stratified by sex 31
Population attributable fraction (PAF) estimations 32
The association between a number of separate psychiatric diagnoses an individual has and reoffending 33
Kaplan-Meier curves for observed violent reoffending in individuals given community sentences stratified by sex and psychiatric disorder 34
Association between individual psychiatric diagnoses and violent reoffending in individuals given community sentences stratified by sex 35
References 36

STROBE reporting checklist

STROBE Statement—Checklist of items that should be included in reports of cohort studies

	Item No	Recommendation	Page No.
Title and abstract	1	(*a*) Indicate the study's design with a commonly used term in the title or the abstract	1
		(*b*) Provide in the abstract an informative and balanced summary of what was done and what was found	1
**Introduction**			
Background/rationale	2	Explain the scientific background and rationale for the investigation being reported	1-2
Objectives	3	State specific objectives, including any prespecified hypotheses	2
**Methods**			
Study design	4	Present key elements of study design early in the paper	2-3
Setting	5	Describe the setting, locations, and relevant dates, including periods of recruitment, exposure, follow-up, and data collection	3
Participants	6	(*a*) Give the eligibility criteria, and the sources and methods of selection of participants. Describe methods of follow-up	3, appendix p 4
		(*b*) For matched studies, give matching criteria and number of exposed and unexposed	NA
Variables	7	Clearly define all outcomes, exposures, predictors, potential confounders, and effect modifiers. Give diagnostic criteria, if applicable	3-5, appendix p 6
Data sources/ measurement	8*	For each variable of interest, give sources of data and details of methods of assessment (measurement). Describe comparability of assessment methods if there is more than one group	3-5
Bias	9	Describe any efforts to address potential sources of bias	3-4
Study size	10	Explain how the study size was arrived at	3, appendix p 5
Quantitative variables	11	Explain how quantitative variables were handled in the analyses. If applicable, describe which groupings were chosen and why	3-4
Statistical methods	12	(*a*) Describe all statistical methods, including those used to control for confounding	4-5
		(*b*) Describe any methods used to examine subgroups and interactions	4-5
		(*c*) Explain how missing data were addressed	3
		(*d*) If applicable, explain how loss to follow-up was addressed	3-4
		(*e*) Describe any sensitivity analyses	3, appendix pp 7-10, 17-19
**Results**			
Participants	13*	(a) Report numbers of individuals at each stage of study—eg numbers potentially eligible, examined for eligibility, confirmed eligible, included in the study, completing follow-up, and analysed	appendix p 5
		(b) Give reasons for non-participation at each stage	3-4, appendix p 5
		(c) Consider use of a flow diagram	appendix p 5
Descriptive data	14*	(a) Give characteristics of study participants (eg demographic, clinical, social) and information on exposures and potential confounders	Table 1
		(b) Indicate number of participants with missing data for each variable of interest	3, appendix p 23
		(c) Summarise follow-up time (eg, average and total amount)	Table 1, appendix p 22
Outcome data	15*	Report numbers of outcome events or summary measures over time	Table 1, appendix p 22
Main results	16	(*a*) Give unadjusted estimates and, if applicable, confounder-adjusted estimates and their precision (eg, 95% confidence interval). Make clear which confounders were adjusted for and why they were included	Figures 1, 2; Tables 2, 3; appendix pp 23-26, 31, 35

		(*b*) Report category boundaries when continuous variables were categorized	Table 1, appendix pp 23-26
		(*c*) If relevant, consider translating estimates of relative risk into absolute risk for a meaningful time period	Table 1
Other analyses	17	Report other analyses done—eg analyses of subgroups and interactions, and sensitivity analyses	appendix pp 9-19, 28-29
**Discussion**			
Key results	18	Summarise key results with reference to study objectives	6-9
Limitations	19	Discuss limitations of the study, taking into account sources of potential bias or imprecision. Discuss both direction and magnitude of any potential bias	9
Interpretation	20	Give a cautious overall interpretation of results considering objectives, limitations, multiplicity of analyses, results from similar studies, and other relevant evidence	6-9
Generalisability	21	Discuss the generalisability (external validity) of the study results	8
**Other information**			
Funding	22	Give the source of funding and the role of the funders for the present study and, if applicable, for the original study on which the present article is based	10

*Give information separately for exposed and unexposed groups.

Note: An Explanation and Elaboration article discusses each checklist item and gives methodological background and published examples of transparent reporting. The STROBE checklist is best used in conjunction with this article (freely available on the Web sites of PLoS Medicine at http://www.plosmedicine.org/, Annals of Internal Medicine at http://www.annals.org/, and Epidemiology at http://www.epidem.com/). Information on the STROBE Initiative is available at http://www.strobe-statement.org.

Additional information about probation in Sweden

Probation in Sweden is the most common community sentence[1]. An individual sentenced to probation is usually supervised for one year. Probation also includes a trial period of three years, which includes the first year of supervision. Probation can also be coupled with conditions, such as treatment (including for substances), vocational training, and community service[2]. Sentenced individuals may also be required to keep the same place of residence. A conditional sentence allows a sentenced individual to avoid a custodial sentence on the condition that they live an orderly life, typically during two year period[2]. Conditional sentences are not supervised. A conditional sentence can be combined with day-fines and include community service. Committing a new crime for any community sentence can result in revocation and imposition of another sentence, such as imprisonment.

Additional notes on methods

Exclusion criteria

We excluded individuals who were born before 1958 because these individuals would not have a complete criminal record in the National Crime Register. We also excluded individuals who committed a crime before the start of the follow-up period but were not sentenced for it by that time, which is known as a pseudo-reconviction. The inclusion of pseudo-reconvictions could have resulted in an overestimation of recidivism risk. Before exclusion of the individuals with identified pseudo-reconvictions, however, we attempted to re-select the starting point of their follow-up period at random again. Individuals whose starting points still implied pseudo-reconvictions after the re-selection were excluded from the analysis.

Selection process for the analysis cohort

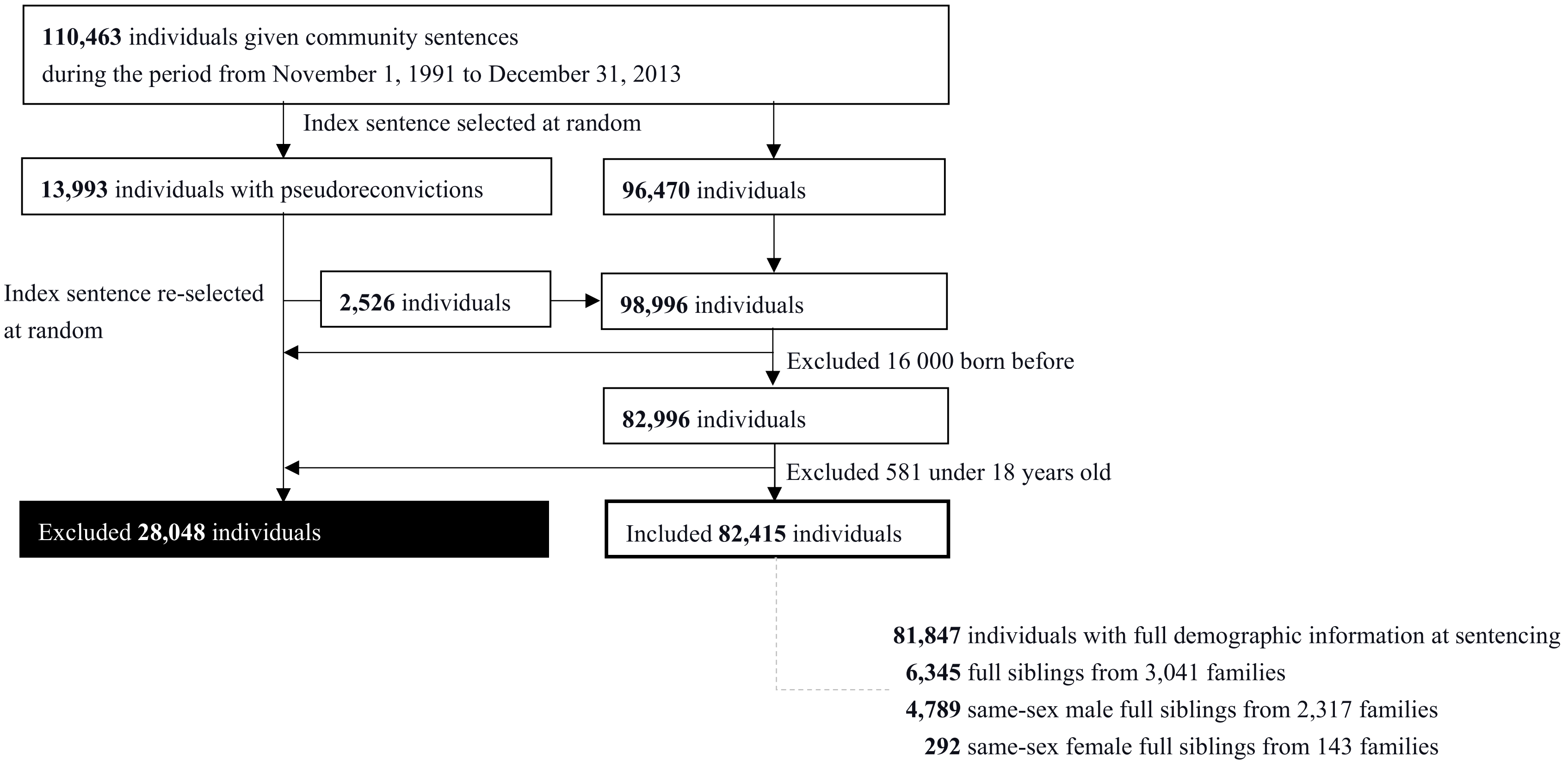

ICD codes for medical history covariates at baseline

Diagnosis	Codes
Any psychiatric	ICD-8: 290-315<br>ICD-9: 290-319<br>ICD-10: F00-F99
Any psychiatric (excluding substance use diagnoses)	-‖- excluding codes for alcohol use and drug use disorders
Schizophrenia spectrum disorder	ICD-8: 295, 297, 298.1-9, 299<br>ICD-9: 295, 297, 298 (ex. A), 299<br>ICD-10: F20-F29
Bipolar disorder	ICD-8: 296.1, 296.3, 296.8<br>ICD-9: 296A, 296C-E, 296W<br>ICD-10: F30-F31
Depressive disorder	ICD-8: 296.2, 296.9, 298.0, 300.4<br>ICD-9: 296B, 296X, 298A, 300E, 311<br>ICD-10: F32-F39
Anxiety disorder	ICD-8: 300 (ex. .4), 305, 307<br>ICD-9: 300 (ex. E), 306, 308, 309<br>ICD-10: F40-F48
Alcohol use disorder	ICD-8: 291, 303<br>ICD-9: 291, 303, 305A<br>ICD-10: F10
Drug use disorder	ICD-8: 304<br>ICD-9: 292, 304, 305 (ex. A)<br>ICD-10: F11-F19
Personality disorder	ICD-8: 301<br>ICD-9: 301 (ex. B)<br>ICD-10: F60-F61
Attention-deficit and hyperactivity disorder	ICD-9: 314<br>ICD-10: F90
Other developmental disorder	ICD-8: 308<br>ICD-9: 299A, 312, 313, 315<br>ICD-10: F80-F98 (ex. F90)
Self-harm	ICD-9: E950-E959<br>ICD-10: X60-X84, Y10-Y34

Results of sibling comparison using sibling pairs discordant by diagnosis

The association were estimated by fitting a Cox regression model in the sample consisting of pairs of same-sex full siblings discordant by a given diagnosis. If an individual had several discordant siblings, then the pair was assigned randomly. All models were additionally adjusted for age.

Association between individual psychiatric diagnoses and general reoffending in same-sex full siblings given community sentences stratified by sex

Outcome: General reoffending

	**Comparison between same-sex full siblings**	
	N of discordant pairs	Hazard ratio (95% CI)
**Men**		
Any psychiatric diagnosis	945	1·55 (1·31-1·83)
Any psychiatric diagnosis (exl. substance use)	761	1·32 (1·09-1·59)
Schizophrenia spectrum	112	1·85 (1·10-3·08)
Bipolar	39	1·38 (0·53-3·61)
Depression	277	0·89 (0·65-1·22)
Anxiety	323	1·33 (1·01-1·76)
Personality disorder	171	1·47 (0·98-2·20)
Attention-deficit hyperactivity	202	0·99 (0·69-1·41)
Other developmental or childhood	191	0·82 (0·58-1·16)
Substance (drug or alcohol) use disorder	766	1·70 (1·41-2·05)
Alcohol use disorder	544	1·37 (1·10-1·69)
Drug use disorder	577	2·02 (1·64-2·49)
**Women**		
Any psychiatric diagnosis	49	2·30 (0·91-5·86)
Any psychiatric diagnosis (exl. substance use)	69	1·87 (0·97-3·61)
Schizophrenia spectrum	8	..
Bipolar	6	..
Depression	43	1·08 (0·50-2·35)
Anxiety	48	1·39 (0·67-2·90)
Personality disorder	28	1·16 (0·49-2·74)
Attention-deficit hyperactivity	11	2·61 (0·36-18·99)
Other developmental or childhood	17	3·43 (0·35-33·29)
Substance (drug or alcohol) use disorder	57	1·73 (0·82-3·65)
Alcohol use disorder	46	0·92 (0·43-1·99)
Drug use disorder	53	1·61 (0·81-3·21)

Note: The estimates were not reported, if number of discordant pairs was less than 10 or the model returned infinite confidence intervals.

[the section continues below]

Association between individual psychiatric diagnoses and violent reoffending in same-sex full siblings given community sentences stratified by sex

Outcome: Violent reoffending

	Comparison between same-sex full siblings	
	N of discordant pairs	Hazard ratio (95% CI)
**Men**		
Any psychiatric diagnosis	945	1·39 (1·05-1·84)
Any psychiatric diagnosis (exl. substance use)	761	1·43 (1·03-1·97)
Schizophrenia spectrum	112	4·57 (1·29-16·18)
Bipolar	39	0·61 (0·10-3·93)
Depression	277	1·02 (0·59-1·74)
Anxiety	323	1·41 (0·83-2·39)
Personality disorder	171	1·78 (0·89-3·57)
Attention-deficit hyperactivity	202	1·26 (0·71-2·22)
Other developmental or childhood	191	1·12 (0·65-1·93)
Substance (drug or alcohol) use disorder	766	1·65 (1·20-2·25)
Alcohol use disorder	544	1·65 (1·15-2·38)
Drug use disorder	577	1·50 (1·04-2·15)
**Women**		
Any psychiatric diagnosis	49	..
Any psychiatric diagnosis (exl. substance use)	69	1·50 (0·33-6·79)
Schizophrenia spectrum	8	..
Bipolar	6	..
Depression	43	..
Anxiety	48	0·25 (0·03-2·25)
Personality disorder	28	3·58 (0·36-35·72)
Attention-deficit hyperactivity	11	..
Other developmental or childhood	17	..
Substance (drug or alcohol) use disorder	57	0·51 (0·13-2·04)
Alcohol use disorder	46	0·91 (0·17-4·87)
Drug use disorder	53	0·20 (0·02-2·44)

Note: The estimates were not reported, if number of discordant pairs was less than 10 or the model returned infinite confidence intervals.

Additional sensitivity analyses

To explore the potential effect of selection in our study, we conducted several additional sensitivity analyses. There are several reasons for conducting the analyses. First, medical data from outpatient visits were only available from 2001 in the National Patient Register. Second, the coverage of outpatient medical data was increasing from 2001 until 2005 and then plateaued (see Murley et al.'s validation study[3]).

Methods

We separated the study cohort into three sub-cohorts by year of the index sentence:

- from 1991 until 2000,
- from 2001 until 2005,
- and from 2006 until 2013.

To explore the contribution of different diagnoses in the potential variability of the sensitivity analyses' results, we explored the association between general and violent reoffending in four diagnostic groups:

1. 'No psychiatric disorders' – having no diagnosis of substance use disorder or other psychiatric disorders.
2. 'Substance use disorder only' – having substance (drug or alcohol) use disorder diagnosis, but no other known psychiatric disorder.
3. 'Psychiatric disorder with comorbid substance use' – having a substance use diagnosis and at least one other psychiatric disorder diagnosis.
4. 'Psychiatric disorder without substance use' – having a psychiatric disorder diagnosis but no known substance use diagnosis.

The association between diagnostic categories and general and violent reoffending were estimated using Cox regression. The diagnostic categories were operationalised as a multilevel categorical variable with 'No psychiatric disorder' as a reference category. All analyses were stratified by sex and adjusted by age. For each outlined sub-cohort, a separate model was fitted. The estimated hazard ratios are presented on the forest plots below (pp 15-16).

We additionally explored the trends in 2-year reoffending rates and sentencing trends in Sweden throughout the study.

Results

Cohort composition

Over the years between 1991 and 2013, the proportion of individuals with substance use diagnosis (single and comorbid) remained withing the range between 22% and 34% without any pronounced trend. However, the proportion of individuals with other psychiatric disorders started to increase after 2001 (Fig. A7-1).

2-year reoffending rates

Both general and violent reoffending decreased substantially from 1991 until 2013 in all sub-cohorts (Fig. A7-2, A7-3). In individuals with substance use diagnosis (single and comorbid), the downward trend in general reoffending rates was less pronounced than in individuals with no substance use disorder diagnosis.

Sentencing trends in Sweden

We used the data from Swedish National Council for Crime Prevention[4] (Fig. A7-4). Over the study period, the number of convictions for property offences decreased from 36,090 in 1991 to 22,547 in 2013. The number of convictions for traffic offences decreased from 41,243 in 1991 to 23,895 in 2013. However, the number of convictions for drug-related offences increased from 4,799 in 1991 to 20,765 in 2013.

Cox regression

In men with substance use diagnoses (single and comorbid), the risk of general and violent reoffending increased over three examined periods. The association between having *substance use disorder only* and general reoffending increased from 1·38 (1·29-1·48) in 1991-2000 to 2·10 (2·01-2·20) in 2006-2013. The association between having a *psychiatric disorder with comorbid substance use* and general reoffending demonstrated a similar trend. The association between having *a psychiatric disorder without substance use* and general reoffending remained stable over time.

In women, the results for general reoffending were similar to the results in men. However, for violent reoffending, the difference in hazard ratios between periods was not significant with large confidence intervals. This was likely due to the small number of violent reoffending cases in women.

Discussion

The association between psychiatric disorders and reoffending varied between selected periods. They were the lowest in the 1991-2000 period and the highest in the 2006-2013 period. Over time, psychiatric disorders became better predictors of both general and violent reoffending.

These findings could be potentially explained by three general trends. First, the overall decrease in recidivism rates over time could reduce the possible ceiling effect for individual recidivism predictors. Second, the introduction of outpatient

data to the Swedish National Patient Register improved the detectability of psychiatric disorders in community-sentenced populations. Third, the number of convictions for drug-related offences quadrupled over the period from 1991 until 2013. This trend, accompanied by the decline in court sentences for other types of offences, could increase the predictive validity of psychiatric disorders for reoffending through the association between substance use diagnosis and drug-related crime.

The findings emphasize the potential mediating role that sentencing and policing practices play as part of the pathway between psychiatric disorders and reconviction in correctional populations. The role of substance use as a predictor for reoffending is likely to be heavily dependent on the country's policies on drug-related crimes, especially use, possession and purchase.

Implication for the primary analyses
Given the results of the sensitivity analyses, the estimates obtained on the primary analysis cohort are likely to be conservative. We additionally provide the main estimates for the 2006-2013 period as the most recent data (see appendix pp 17-19).

[See figures below on pp 11-16]

Figure A7-1. The proportion of individuals with psychiatric disorders in the total cohort by year of release

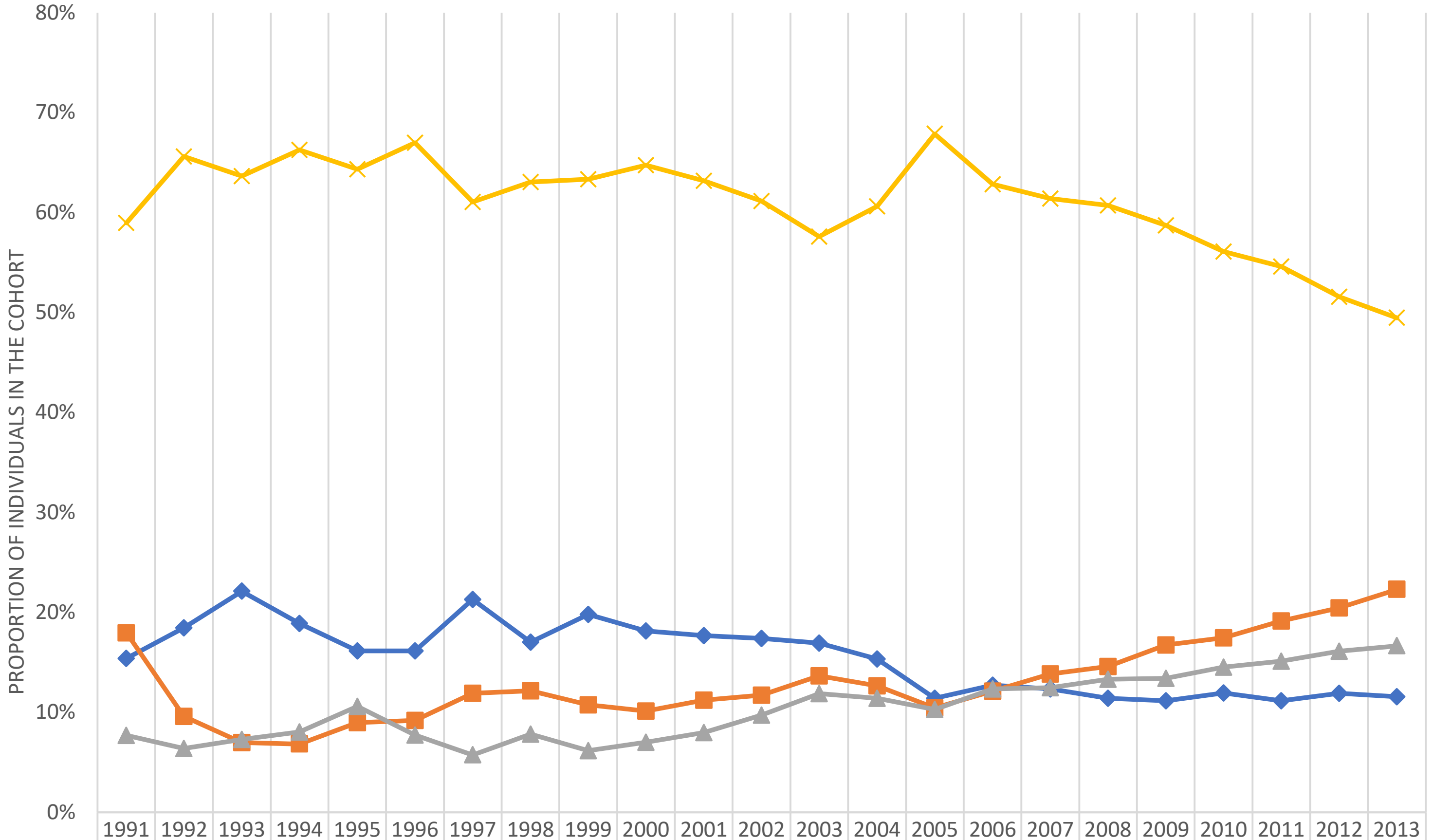

	1991	1992	1993	1994	1995	1996	1997	1998	1999	2000	2001	2002	2003	2004	2005	2006	2007	2008	2009	2010	2011	2012	2013
Substance use disorder only	15%	18%	22%	19%	16%	16%	21%	17%	20%	18%	18%	17%	17%	15%	11%	13%	12%	11%	11%	12%	11%	12%	12%
Psychiatric disorder WITH comorbid substance use	18%	10%	7%	7%	9%	9%	12%	12%	11%	10%	11%	12%	14%	13%	10%	12%	14%	15%	17%	17%	19%	20%	22%
Psychiatric disorder WITHOUT substance use	8%	6%	7%	8%	11%	8%	6%	8%	6%	7%	8%	10%	12%	11%	10%	12%	12%	13%	13%	15%	15%	16%	17%
No psychiatric disorders	59%	66%	64%	66%	64%	67%	61%	63%	63%	65%	63%	61%	58%	61%	68%	63%	61%	61%	59%	56%	55%	52%	49%

YEAR OF SENTENCE

Figure A7-2. The 2-year general reoffending rates in individuals with psychiatric disorders in the total cohort by year of release

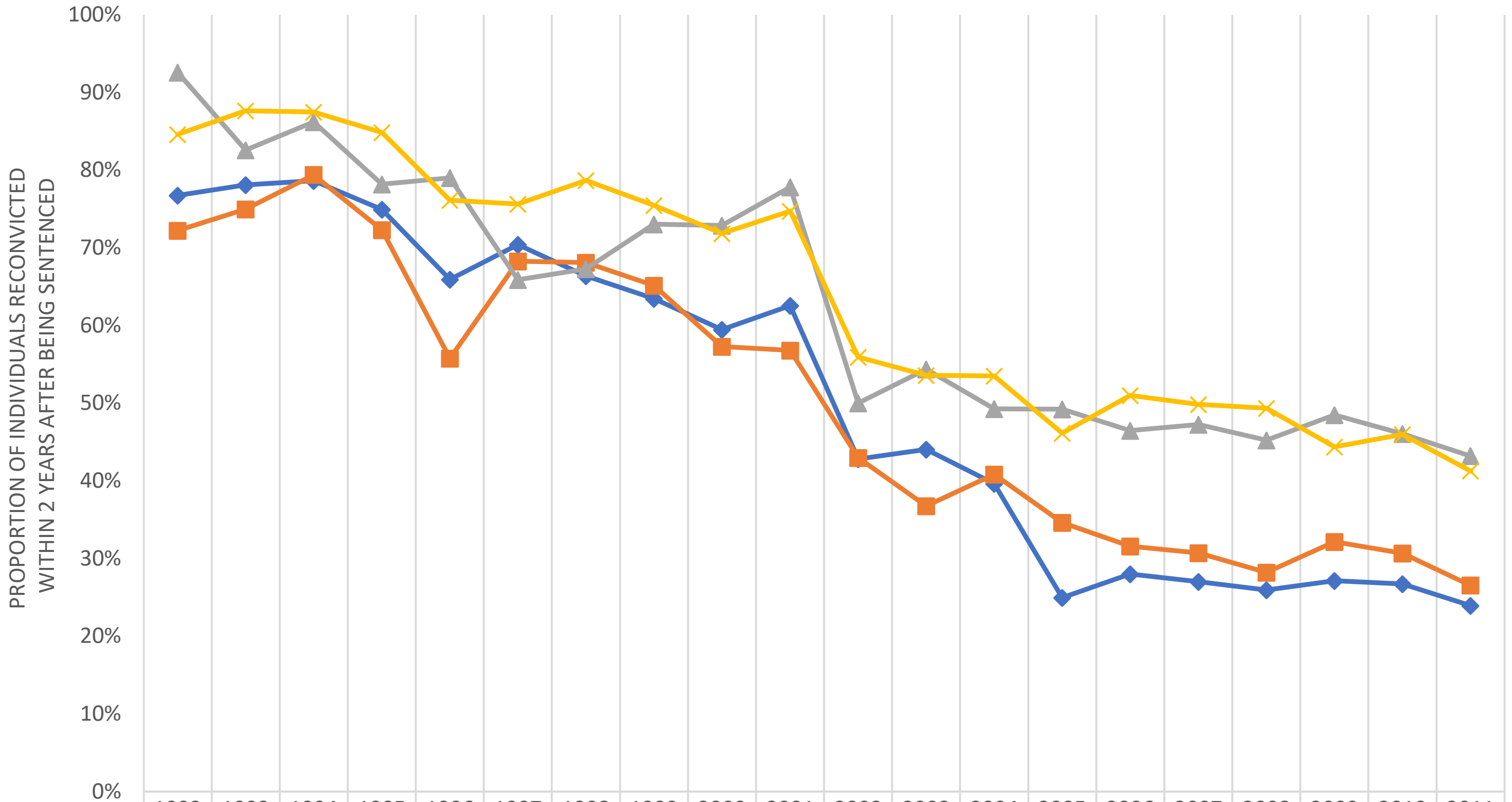

	1992	1993	1994	1995	1996	1997	1998	1999	2000	2001	2002	2003	2004	2005	2006	2007	2008	2009	2010	2011
Substance use disorder only	77%	78%	79%	75%	66%	70%	66%	63%	59%	63%	43%	44%	40%	25%	28%	27%	26%	27%	27%	24%
Psychiatric disorder WITH comorbid substance use	72%	75%	79%	72%	56%	68%	68%	65%	57%	57%	43%	37%	41%	35%	32%	31%	28%	32%	31%	27%
Psychiatric disorder WITHOUT substance use	93%	83%	86%	78%	79%	66%	67%	73%	73%	78%	50%	54%	49%	49%	46%	47%	45%	48%	46%	43%
No psychiatric disorders	85%	88%	88%	85%	76%	76%	79%	75%	72%	75%	56%	54%	54%	46%	51%	50%	49%	44%	46%	41%

YEAR OF SENTENCE

Figure A7-3. The 2-year violent reoffending rates in individuals with psychiatric disorders in the total cohort by year of release

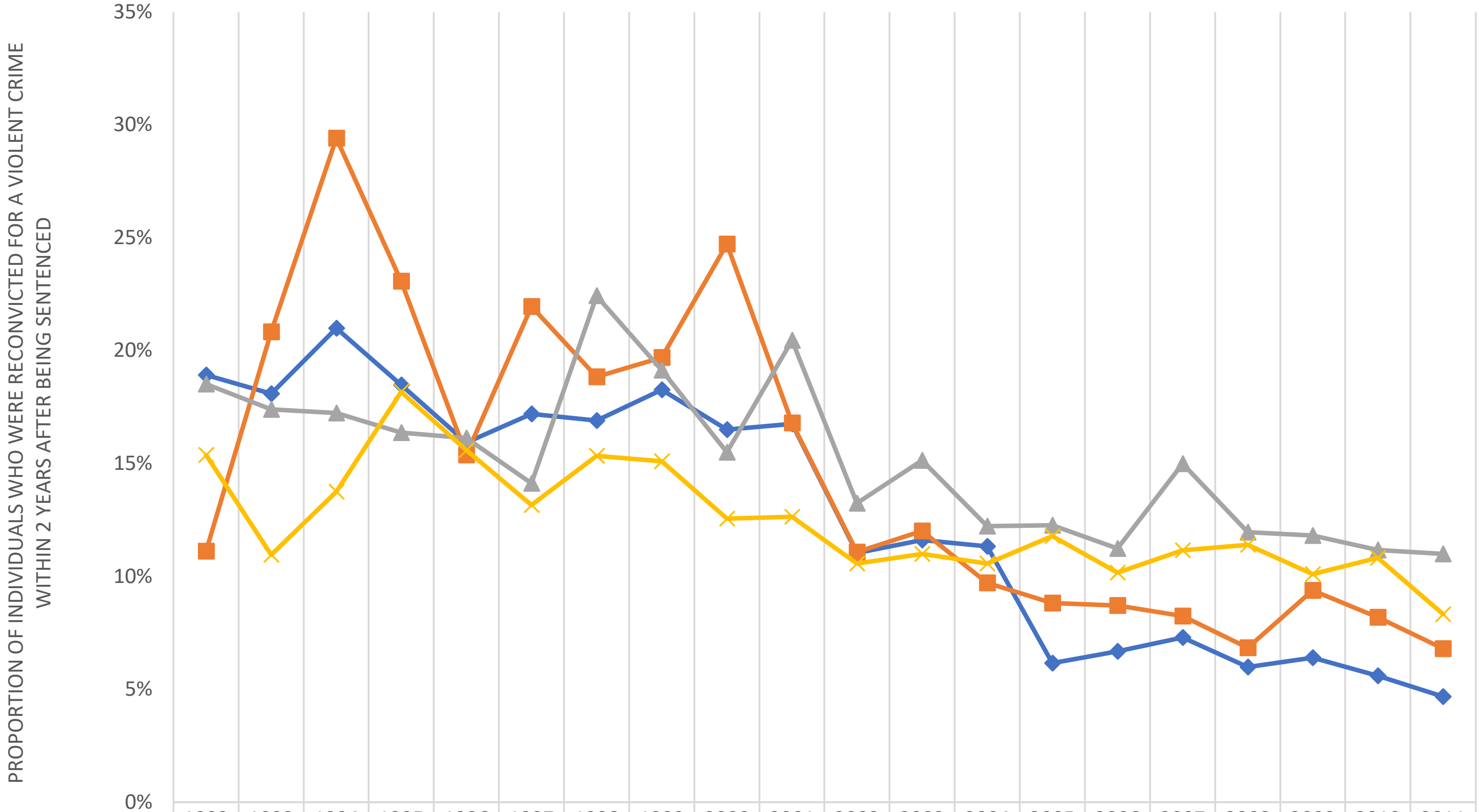

	1992	1993	1994	1995	1996	1997	1998	1999	2000	2001	2002	2003	2004	2005	2006	2007	2008	2009	2010	2011
Substance use disorder only	19%	18%	21%	18%	16%	17%	17%	18%	17%	17%	11%	12%	11%	6%	7%	7%	6%	6%	6%	5%
Psychiatric disorder WITH comorbid substance use	11%	21%	29%	23%	15%	22%	19%	20%	25%	17%	11%	12%	10%	9%	9%	8%	7%	9%	8%	7%
Psychiatric disorder WITHOUT substance use	19%	17%	17%	16%	16%	14%	22%	19%	16%	20%	13%	15%	12%	12%	11%	15%	12%	12%	11%	11%
No psychiatric disorders	15%	11%	14%	18%	16%	13%	15%	15%	13%	13%	11%	11%	11%	12%	10%	11%	11%	10%	11%	8%

YEAR OF SENTENCE

Figure A7-4. Number of convictions handed down in Sweden by the type of crime and year of sentence

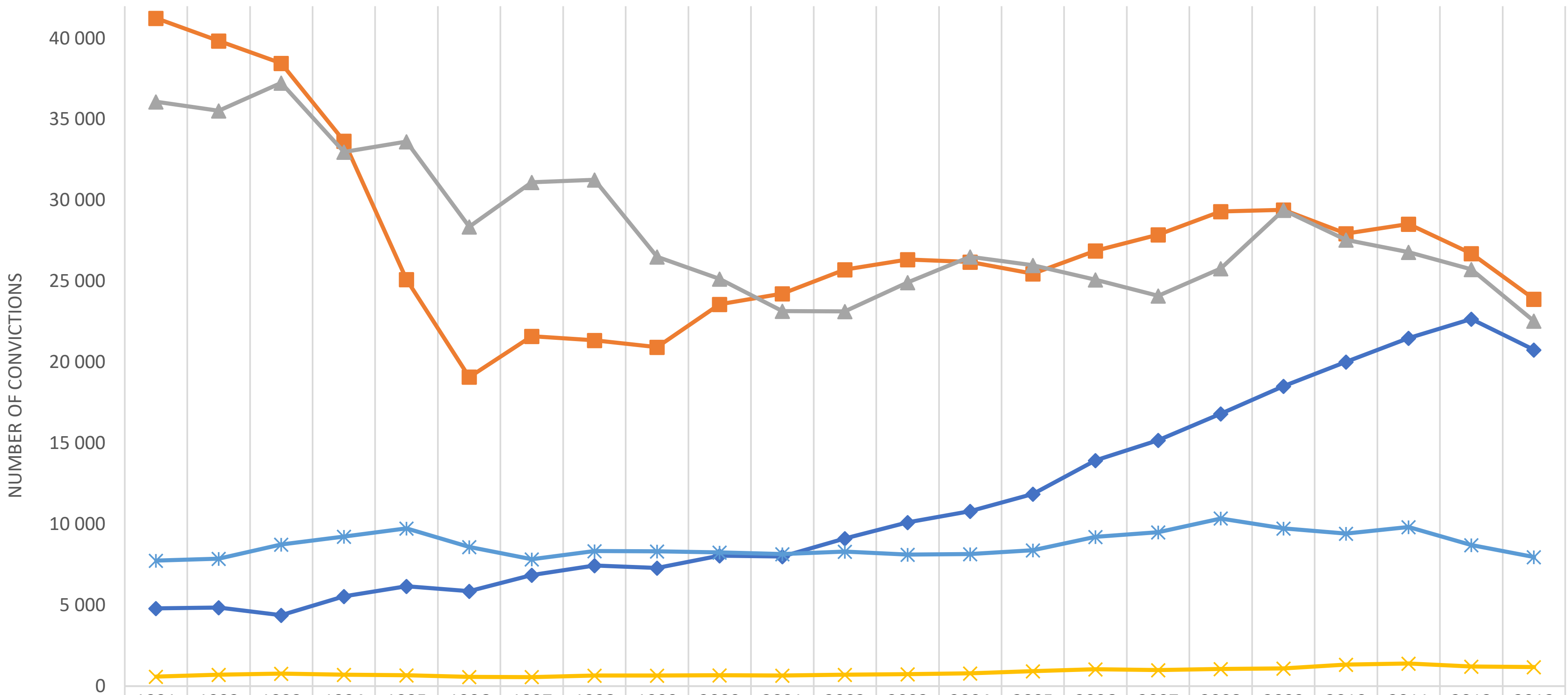

	1991	1992	1993	1994	1995	1996	1997	1998	1999	2000	2001	2002	2003	2004	2005	2006	2007	2008	2009	2010	2011	2012	2013
Drug-relate crime	4 799	4 854	4 382	5 544	6 164	5 862	6 859	7 441	7 291	8 055	8 005	9 121	10 106	10 808	11 862	13 932	15 179	16 817	18 525	20 021	21 482	22 672	20 765
Traffic crime	41 243	39 844	38 468	33 665	25 115	19 091	21 613	21 350	20 923	23 574	24 234	25 717	26 338	26 189	25 460	26 881	27 877	29 302	29 416	27 940	28 536	26 719	23 895
Property crimes	36 090	35 523	37 249	32 989	33 618	28 358	31 110	31 256	26 516	25 144	23 157	23 137	24 933	26 515	25 981	25 098	24 099	25 784	29 393	27 563	26 789	25 728	22 547
Sexual offences	585	707	764	698	666	576	567	650	652	675	658	702	731	793	917	1 038	985	1 055	1 090	1 324	1 398	1 217	1 167
Against health	7 749	7 869	8 747	9 231	9 745	8 597	7 835	8 340	8 322	8 253	8 157	8 307	8 120	8 150	8 389	9 210	9 497	10 354	9 744	9 424	9 819	8 703	7 975

YEAR OF SENTENCE

Note: data obtained from Swedish National Council for Crime Prevention[4].

Figure A7-5. The association between psychiatric disorders and general reoffending by sex and year of release

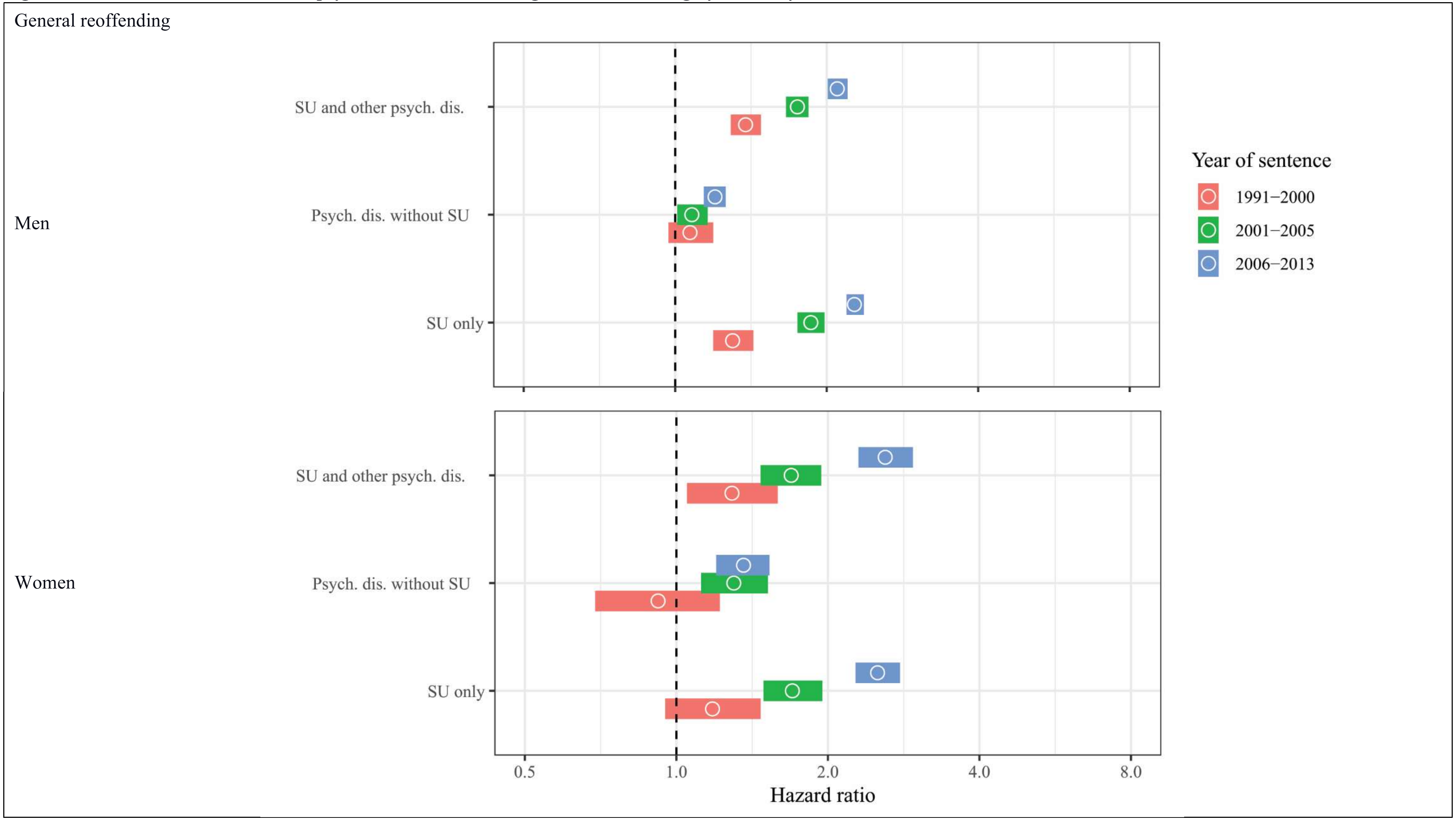

Note: SU and other psych. dis. – psychiatric disorder with comorbid substance use. Psych. dis. without SU – psychiatric disorder without substance use. SU only – substance use disorder only.

Figure A7-6. The association between psychiatric disorders and violent reoffending by sex and year of release

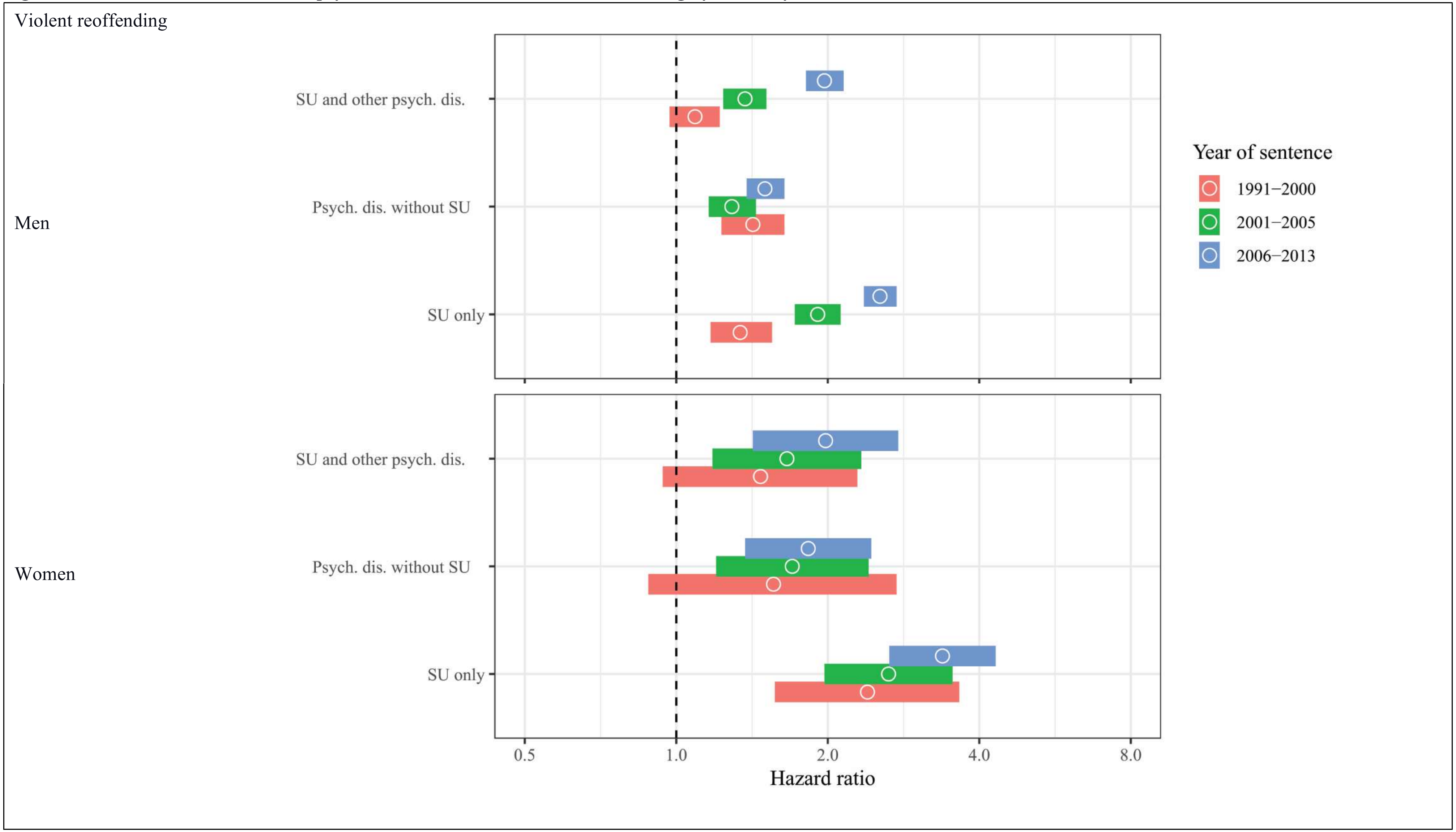

Note: SU and other psych. dis. – psychiatric disorder with comorbid substance use. Psych. dis. without SU – psychiatric disorder without substance use. SU only – substance use disorder only.

The analysis for the sub-cohort of individuals sentenced in the period from 2006 to 2013

Baseline characteristics and follow-up data of adult individuals with same-sex full siblings receiving community sentences from November 1, 1991, to December 31, 2013

	Men	Women	Total
Number of individuals	49,354 (85·3%)	8,534 (14·7%)	57,888 (100·0%)
**Baseline**			
Median age	28 (IQR: 22-39)	31 (IQR: 23-42)	28 (IQR: 22-40)
Married or in a registered partnership	5,721 (11·6%)	1,210 (14·2%)	6,931 (12·0%)
Employed	23,632 (47·9%)	3,088 (36·2%)	26,720 (46·2%)
Years of education			
< 9 yr	1,761 (3·6%)	372 (4·4%)	2,133 (3·7%)
9-11 yr	42,001 (85·1%)	6,963 (81·6%)	48,964 (84·6%)
≥ 12 yr	3,842 (7·8%)	941 (11·0%)	4,783 (8·3%)
Recipient of income support	14,550 (29·5%)	3,689 (43·2%)	18,239 (31·5%)
Prior criminal history	35,733 (72·4%)	5,329 (62·4%)	41062 (70·9%)
Prior violent crime	16,913 (34·3%)	1,624 (19·0%)	18,537 (32·0%)
Prior imprisonment	9,702 (19·7%)	855 (10·0%)	10,557 (18·2%)
Index violent offence	25,529 (51·7%)	3,283 (38·5%)	28,812 (49·8%)
Any psychiatric disorder	19,903 (40·3%)	5,257 (61·6%)	25,160 (43·5%)
Any psychiatric disorder (excluding substance use)	14,079 (28·5%)	4,291 (50·3%)	18,370 (31·7%)
Schizophrenia spectrum disorder	1,470 (3·0%)	416 (4·9%)	1,886 (3·3%)
Bipolar disorder	596 (1·2%)	310 (3·6%)	906 (1·6%)
Depression	4,494 (9·1%)	1,650 (19·3%)	6,144 (10·6%)
Anxiety disorder	4,356 (8·8%)	1,434 (16·8%)	5,790 (10·0%)
Personality disorder	1,807 (3·7%)	984 (11·5%)	2,791 (4·8%)
Attention-deficit hyperactivity disorder	3,151 (6·4%)	586 (6·9%)	3,737 (6·5%)
Other developmental or childhood disorder	2,544 (5·2%)	615 (7·2%)	3,159 (5·5%)
Substance (drug or alcohol) use disorder	13,341 (27·0%)	3,503 (41·0%)	16,844 (29·1%)
Alcohol use disorder	8,489 (17·2%)	2,255 (26·4%)	10,744 (18·6%)
Drug use disorder	8,242 (16·7%)	2,352 (27·6%)	10,594 (18·3%)
**Follow-up data. General reoffending**			
Number of persons-year at risk	123,898·6	23,389·9	147,288·5
Incidents of reoffending during follow-up	17,445 (35·3%)	2,386 (28·0%)	19,831 (34·3%)
Median time to any new offence (months)			
All individuals	22·3 (IQR: 8-47·9)	26·2 (IQR: 9·2-51·9)	22·9 (IQR: 8·1-48·7)
Individuals with psychiatric disorder	15·7 (IQR: 5·4-37·5)	21·8 (IQR: 6·9-46)	17·0 (IQR: 5·7-38·9)
Individual without psychiatric disorder	27·3 (IQR: 10·3-54·5)	34·7 (IQR: 14·1-60·9)	27·8 (IQR: 10·6-55·2)
Reoffending rate (cumulative)			
1-year	10,167 (20·6%)	1,462 (17·1%)	11,629 (20·1%)
2-year	13,883 (28·1%)	1,919 (22·5%)	15,802 (27·3%)
3-year	15,612 (31·6%)	2,145 (25·1%)	17,757 (30·7%)
4-year	16,514 (33·5%)	2,274 (26·6%)	18,788 (32·5%)
5-year	17,036 (34·5%)	2,343 (27·5%)	19,379 (33·5%)
Died during follow-up	753 (1·5%)	116 (1·4%)	869 (1·5%)
Emigrated during follow-up	572 (1·2%)	71 (0·8%)	643 (1·1%)

**Follow-up data. Violent reoffending**			
Number of persons-year at risk	155,292·2	28,838	184,130·2
Incidents of reoffending during follow-up	4,619 (9·4%)	442 (5·2%)	5,061 (8·7%)
Median time to a violent offence (months)			
All individuals	33·4 (IQR: 13·8-58·6)	37·3 (IQR: 16·3-61·8)	33·9 (IQR: 14·2-59·1)
Individuals with psychiatric disorder	26·4 (IQR: 10·4-50·4)	33·8 (IQR: 14·5-58·1)	27·6 (IQR: 11-51·9)
Individual without psychiatric disorder	38 (IQR: 16·8-62·8)	43·3 (IQR: 21·5-67·9)	38·4 (IQR: 17·6-63·1)
Reoffending rate (cumulative)			
1-year	2,211 (4·5%)	209 (2·4%)	2,420 (4·2%)
2-year	3,353 (6·8%)	310 (3·6%)	3,663 (6·3%)
3-year	3,946 (8·0%)	364 (4·3%)	4,310 (7·4%)
4-year	4,288 (8·7%)	410 (4·8%)	4,698 (8·1%)
5-year	4,469 (9·1%)	431 (5·1%)	4,900 (8·5%)
Imprisoned during follow-up	3,504 (7·1%)	358 (4·2%)	3,862 (6·7%)
Died during follow-up	1,100 (2·2%)	191 (2·2%)	1,291 (2·2%)
Emigrated during follow-up	743 (1·5%)	89 (1·0%)	832 (1·4%)

[the section continues below]

Association between individual psychiatric diagnoses and reoffending in individuals given community sentences in 2006-2013 stratified by sex (all models adjusted for age)

		Hazard ratio (95% CI)	
	N with diagnosis	General reoffending	Violent reoffending
**Men (N = 49,354)**			
Any psychiatric diagnosis	19,903	1·80 (1·75-1·86)	1·98 (1·87-2·10)
Any psychiatric diagnosis (excl. substance use)	14,079	1·47 (1·43-1·52)	1·75 (1·65-1·86)
Schizophrenia spectrum	1,470	1·57 (1·44-1·70)	2·17 (1·90-2·49)
Bipolar	596	1·21 (1·05-1·39)	1·39 (1·08-1·81)
Depression	4,494	1·19 (1·13-1·25)	1·29 (1·17-1·42)
Anxiety	4,356	1·34 (1·27-1·40)	1·38 (1·26-1·52)
Personality disorder	1,807	1·86 (1·74-2·00)	2·42 (2·14-2·73)
Attention-deficit hyperactivity	3,151	1·69 (1·60-1·78)	2·10 (1·91-2·31)
Other developmental or childhood	2,544	1·38 (1·30-1·47)	1·95 (1·76-2·16)
Substance (drug or alcohol) use disorder	13,341	2·12 (2·06-2·19)	2·10 (1·97-2·23)
Alcohol use disorder	8,489	1·60 (1·54-1·66)	1·98 (1·85-2·12)
Drug use disorder	8,242	2·57 (2·48-2·66)	2·14 (2·00-2·29)
**Women (N = 8,534)**			
Any psychiatric diagnosis	5,257	2·09 (1·91-2·29)	2·54 (2·03-3·18)
Any psychiatric diagnosis (excl. substance use)	4,291	1·51 (1·39-1·63)	2·22 (1·83-2·71)
Schizophrenia spectrum	416	1·54 (1·30-1·81)	2·90 (2·14-3·91)
Bipolar	310	0·98 (0·78-1·23)	1·33 (0·83-2·14)
Depression	1,650	1·21 (1·10-1·34)	1·14 (0·90-1·45)
Anxiety	1,434	1·14 (1·03-1·27)	1·52 (1·21-1·90)
Personality disorder	984	1·58 (1·41-1·76)	2·52 (2·01-3·16)
Attention-deficit hyperactivity	586	1·55 (1·34-1·80)	1·92 (1·41-2·61)
Other developmental or childhood	615	1·61 (1·40-1·84)	2·51 (1·94-3·26)
Substance (drug or alcohol) use disorder	3,503	2·27 (2·09-2·46)	2·31 (1·91-2·80)
Alcohol use disorder	2,255	1·30 (1·19-1·42)	2·19 (1·81-2·66)
Drug use disorder	2,352	2·84 (2·62-3·08)	2·00 (1·66-2·42)

Formula and R code for sibling comparison

The stratified Cox regression model were fitted into a sub cohort of same-sex siblings using the following formula:

$$\lambda_{if}(t) = \lambda_{0f}(t) exp\left(\beta_1 \cdot Age_i + \beta_2 \cdot Diagnosis_i\right)$$

where $\lambda_{ij}(t)$ is the hazard function for the i-th individual, who belongs to the f-th family; $\lambda_{0f}(t)$ is the baseline hazard for the f-th family; β1, β2 are the regression parameters; $Diagnosis_i$ is a binary variable for a given individual diagnosis; Age_i is a continuous variable, age in years. Consequently, each family has its own baseline hazard that absorbs any confounding shared between siblings. The model assumes the constant effect of measured factors and unmeasured shared familial factors over time.

Data structure

familyID	ID	REC	Time_stop	Sex	Age	Any_diagnosis	SCH	SU	..
123123	111	1	183	Male	20	1	1	0	..
123123	222	0	307	Male	29	0	0	0	..
252525	333	1	249	Male	31	0	0	0	..
252525	444	0	100	Male	34	1	0	1	..

- familyID – the code unique to each sibship,
- ID – the individual code,
- REC – the outcome (general or violent reoffending),
- Time_stop – the time in days from the start of the follow-up period until the outcome or censoring event,
- Sex – biological sex (men and women were analysed separately),
- Age – age in years,
- Any_diagnosis – a variable representing a history of any prior psychiatric diagnosis,
- SCH – a variable representing a diagnosis of schizophrenia before the index sentence.
- SU – a variable representing a diagnosis of drug or alcohol use disorder before the index sentence.
- .. – other columns with covariates.

R code for analysis loop

```
library(dplyr)
library(haven)
library(survival)

risk_factors <- c("Any_diagnosis", "SCH", "SU")
data <- read_dta(paste0('path')) #path to the .dta file with data (data organized as shown above)

for (factor in unique(risk_factors)) {
cox <- coxph(Surv(Time_stop, REC) ~ eval(parse(text=factor)) + Age + strata(familyID),
        data = data, method = 'breslow')
summary(cox)
}
```

R code for population attributable fractions (PAF)

Data structure

ID	REC	Time_stop	Sex	Age	SU	..
111	1	183	Male	20	0	..
222	0	307	Male	29	0	..
333	1	249	Male	31	0	..
444	0	100	Male	34	1	..

- ID – the individual code,
- REC – the outcome (general or violent reoffending),
- Time_stop – the time in days from the start of the follow-up period until the outcome or censoring event,
- Sex – biological sex (men and women were analysed separately),
- Age – age in years,
- SU – a variable representing a diagnosis of drug or alcohol use disorder before the index sentence.
- .. - other columns with covariates.

R code for analysis

```
library(AF)
library(dplyr)
library(survival)

data <- read_dta(paste0('path')) #path to the .dta file with data (data organized as shown above)
data_men <- filter(data, Sex == 'Male')

#example with the substance use (SU) as exposure
cox <- coxph(Surv(time_stop, REC) ~ SU + Age, data=data_men, ties="breslow")
summary(cox)

times <- c(182, 365, 730, 1095, 8071) #setting time intervals for PAF in days
men_AF <- AFcoxph(cox, data=data_men, exposure = 'SU', times=times)
summary(men_AF)
```

Kaplan-Meier curves for obversed general and violent reoffending in the full cohort

Kaplan-Meier curve for **general reoffending** in individuals given community sentences.

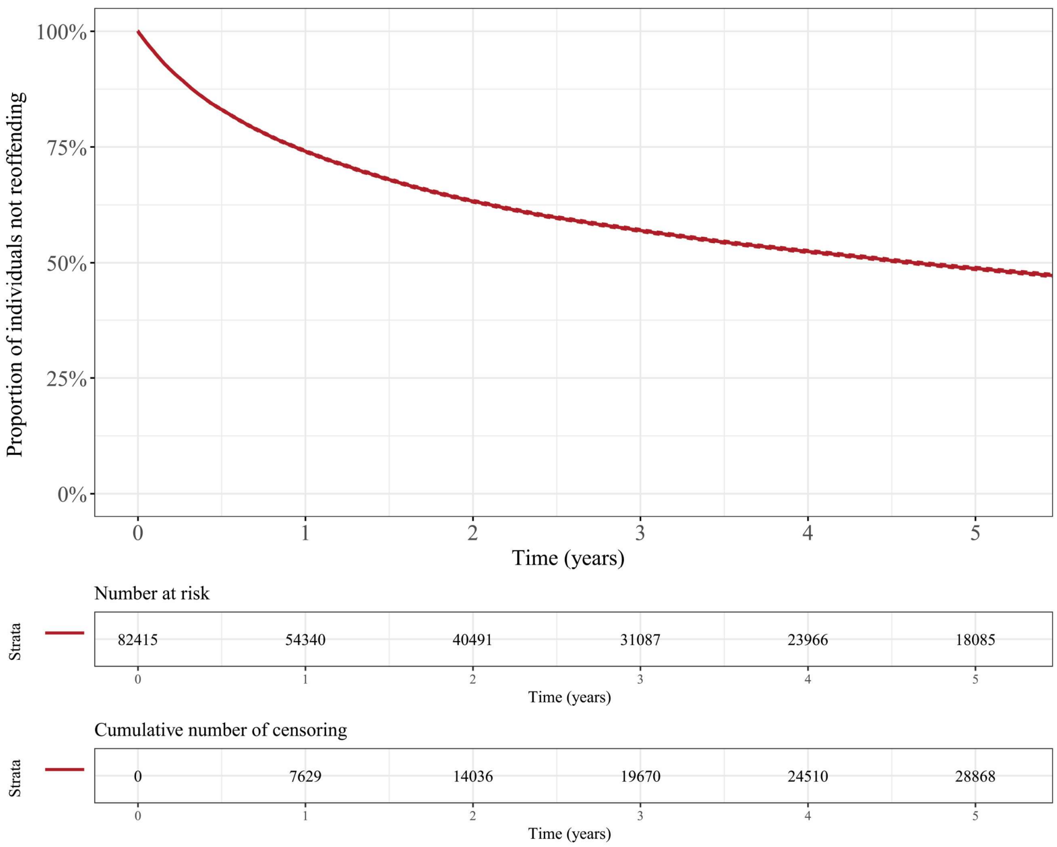

Kaplan-Meier curve for **violent reoffending** in individuals given community sentences.

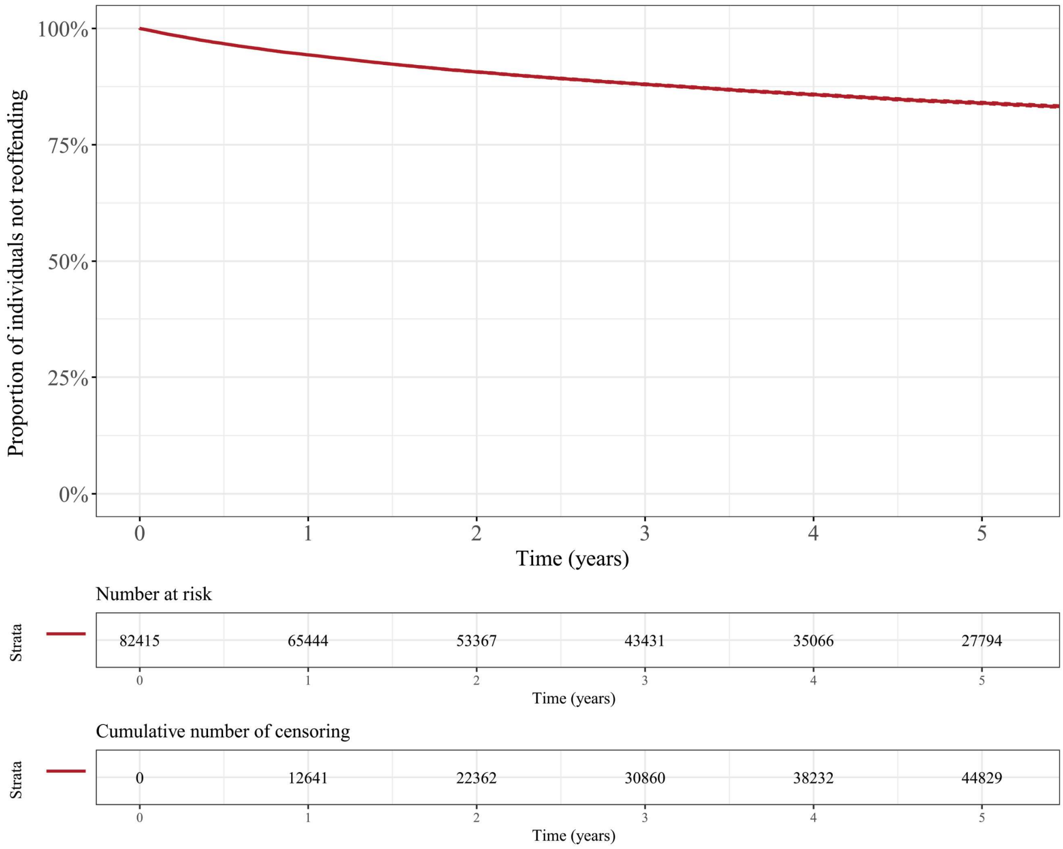

Univariate analysis

Unadjusted association between baseline sociodemographic/clinical factor and reoffending (general and violent) in men given community sentences

Missing data: 510 men have missing values for marital status, employment, and income support. 2,912 men have missing values for education.

Men (N = 70,643)		General reoffending		Violent reoffending	
	N of individuals	N with outcome	Hazard ratio (95% CI)	N with outcome	Hazard ratio (95% CI)
**Age**					
18-24 years	28,299	15,560 (55·0%)	1	5,400 (19·1%)	1
25-39 years	27,468	13,101 (47·7%)	0·78 (0·76-0·80)	3,892 (14·2%)	0·70 (0·67-0·73)
≥ 40 years	14,876	5,113 (34·4%)	0·55 (0·54-0·57)	1,299 (8·7%)	0·46 (0·43-0·49)
**Civil status**					
Single	7,453	2,464 (33·1%)	0·59 (0·57-0·62)	719 (9·6%)	0·6 (0·56-0·65)
Married	62,680	31,100 (49·6%)	1	9,824 (15·7%)	1
**Years of education**					
< 9 yr	2,322	1,237 (53·3%)	1	360 (15·5%)	1
9-12 yr	60,649	29,594 (48·8%)	0·78 (0·74-0·83)	320 (6·7%)	0·35 (0·30-0·41)
> 12 yr	4,760	1,153 (24·2%)	0·33 (0·30-0·36)	9,240 (15·2%)	0·84 (0·75-0·93)
**Employed**					
Yes	31,153	10,625 (34·1%)	0·45 (0·44-0·46)	3,240 (10·4%)	0·49 (0·47-0·51)
No	38,980	22,939 (58·8%)	1	7,303 (18·7%)	1
**Recipient of income support**					
Yes	24,367	15,896 (65·2%)	2·21 (2·16-2·26)	5,126 (21·0%)	1·97 (1·90-2·05)
No	45,766	17,668 (38·6%)	1	5,417 (11·8%)	1
**Prior criminal history**					
Any prior convictions	54,395	29,681 (54·6%)	2·72 (2·63-2·81)	9,369 (17·2%)	2·48 (2·34-2·63)
No prior convictions	16,248	4,093 (25·2%)	1	1,222 (7·5%)	1
**Prior violent crime**					
Prior conviction for a violent offence	27,222	16,600 (61·0%)	1·9 (1·86-1·94)	6,147 (22·6%)	2·54 (2·45-2·64)
No convictions for a violent offence	43,421	17,174 (39·6%)	1	4,444 (10·2%)	1
**Prior prison**					
Prior imprisonment	15,755	10,149 (64·4%)	1·9 (1·86-1·95)	2,906 (18·4%)	1·54 (1·47-1·60)
No prior imprisonment	54,888	23,625 (43·0%)	1	7,685 (14·0%)	1
**Index violent crime**					
Yes	32,941	13,943 (42·3%)	0·77 (0·76-0·79)	5,881 (17·9%)	1·54 (1·48-1·60)
No	37,702	19,831 (52·6%)	1	4,710 (12·5%)	1
**Any psychiatric disorder**					
Yes	27,138	14,281 (52·6%)	1·46 (1·43-1·5)	4,536 (16·7%)	1·43 (1·38-1·49)
No	43,505	19,493 (44·8%)	1	6,055 (13·9%)	1
**Any psychiatric disorder (excl. substance use)**					
Yes	18,047	8,717 (48·3%)	1·19 (1·16-1·22)	2,964 (16·4%)	1·34 (1·28-1·40)
No	52,596	25,057 (47·6%)	1	7,627 (14·5%)	1
**Schizophrenia spectrum disorder**					
Yes	2,032	1,039 (51·1%)	1·21 (1·14-1·29)	425 (20·9%)	1·55 (1·40-1·70)
No	68,611	32,735 (47·7%)	1	10,166 (14·8%)	1

**Bipolar disorder**					
Yes	690	266 (38·6%)	0·90 (0·80-1·01)	82 (11·9%)	0·95 (0·76-1·18)
No	69,953	33,508 (47·9%)	1	10,509 (15·0%)	1
**Depressive disorder**					
Yes	5,447	2,308 (42·4%)	0·95 (0·91-0·99)	720 (13·2%)	0·98 (0·91-1·05)
No	65,196	31,466 (48·3%)	1	9,871 (15·1%)	1
**Anxiety disorder**					
Yes	5,604	2,699 (48·2%)	1·12 (1·08-1·17)	875 (15·6%)	1·17 (1·09-1·25)
No	65,039	31,075 (47·8%)	1	9,716 (14·9%)	1
**Alcohol use disorder**					
Yes	11,569	6,101 (52·7%)	1·32 (1·28-1·36)	2,091 (18·1%)	1·44 (1·37-1·51)
No	59,074	27,673 (46·8%)	1	8,500 (14·4%)	1
**Drug use disorder**					
Yes	11,864	7,607 (64·1%)	2·12 (2·06-2·17)	2,144 (18·1%)	1·57 (1·50-1·65)
No	58,779	26,167 (44·5%)	1	8,447 (14·4%)	1
**Substance use disorder (alcohol or drug use disorder)**					
Yes	18,680	10,777 (57·7%)	1·71 (1·67-1·75)	3,272 (17·5%)	1·49 (1·43-1·56)
No	51,963	22,997 (44·3%)	1	7,319 (14·1%)	1
**Personality disorder**					
Yes	2,671	1,578 (59·1%)	1·52 (1·45-1·6)	606 (22·7%)	1·78 (1·64-1·93)
No	67,972	32,196 (47·4%)	1	9,985 (14·7%)	1
**Attention deficit hyperactivity disorder**					
Yes	3,370	1,594 (47·3%)	1·45 (1·38-1·52)	568 (16·9%)	1·75 (1·61-1·9)
No	67,273	32,180 (47·8%)	1	10,023 (14·9%)	1
**Other developmental or childhood disorder**					
Yes	3,246	1,673 (51·5%)	1·37 (1·31-1·44)	684 (21·1%)	1·83 (1·7-1·98)
No	67,397	32,101 (47·6%)	1	9,907 (14·7%)	1
**Prior self-harm**					
Yes	5,811	2,951 (50·8%)	1·26 (1·22-1·31)	979 (16·8%)	1·34 (1·25-1·43)
No	64,832	30,823 (47·5%)	1	9,612 (14·8%)	1

[the section continues below]

Unadjusted association between baseline sociodemographic/clinical factor and reoffending (general and violent) in women given community sentences.

Missing data: 58 women have missing values for marital status, employment, and income support. 423 women have missing values for education.

**Women (N = 11,772)**		**General reoffending**		**Violent reoffending**	
	N of individuals	N with outcome	Hazard ratio (95% CI)	N with outcome	Hazard ratio (95% CI)
**Age**					
18-24 years	3,616	1,311 (36·3%)	1	322 (8·9%)	1
25-39 years	4,815	2,068 (42·9%)	1·22 (1·13-1·30)	390 (8·1%)	0·89 (0·77-1·03)
≥ 40 years	3,341	1,055 (31·6%)	0·89 (0·82-0·96)	156 (4·7%)	0·57 (0·47-0·70)
**Civil status**					
Single	1,642	557 (33·9%)	0·85 (0·78-0·93)	106 (6·5%)	0·85 (0·69-1·04)
Married	10,072	3,856 (38·3%)	1	759 (7·5%)	1
**Years of education**					
< 9 yr	500	231 (46·2%)	1	50 (10·0%)	1
9-12 yr	9,705	3,720 (38·3%)	0·73 (0·64-0·83)	721 (7·4%)	0·67 (0·51-0·90)
> 12 yr	1,144	265 (23·2%)	0·41 (0·35-0·49)	46 (4·0%)	0·38 (0·26-0·57)
**Employed**					
Yes	3,909	826 (21·1%)	0·38 (0·35-0·41)	151 (3·9%)	0·41 (0·34-0·49)
No	7,805	3,587 (46·0%)	1	714 (9·1%)	1
**Recipient of income support**					
Yes	5,634	2,847 (50·5%)	2·3 (2·16-2·45)	562 (10·0%)	1·96 (1·7-2·25)
No	6,080	1,566 (25·8%)	1	303 (5·0%)	1
**Prior criminal history**					
Any prior convictions	7,832	3,728 (47·6%)	3·32 (3·06-3·6)	694 (8·9%)	2·04 (1·72-2·4)
No prior convictions	3,940	706 (17·9%)	1	174 (4·4%)	1
**Prior violent crime**					
Prior conviction for a violent offence	2,373	1,190 (50·1%)	1·69 (1·58-1·81)	345 (14·5%)	2·95 (2·58-3·38)
No convictions for a violent offence	9,399	3,244 (34·5%)	1	523 (5·6%)	1
**Prior prison**					
Prior imprisonment	1,392	873 (62·7%)	2·39 (2·22-2·58)	114 (8·2%)	1·24 (1·02-1·51)
No prior imprisonment	10,380	3,561 (34·3%)	1	754 (7·3%)	1
**Index violent crime**					
Yes	4,013	1,178 (29·4%)	0·69 (0·65-0·74)	461 (11·5%)	2·61 (2·28-2·99)
No	7,759	3,256 (42·0%)	1	407 (5·2%)	1
**Any psychiatric disorder**					
Yes	7,062	2,986 (42·3%)	1·68 (1·58-1·79)	632 (8·9%)	2·07 (1·78-2·40)
No	4,710	1,448 (30·7%)	1	236 (5·0%)	1
**Any psychiatric disorder (excl. substance use)**					
Yes	5,486	2,150 (39·2%)	1·25 (1·18-1·33)	494 (9·0%)	1·81 (1·58-2·08)
No	6,286	2,284 (36·3%)	1	374 (5·9%)	1
**Schizophrenia spectrum disorder**					
Yes	563	247 (43·9%)	1·29 (1·13-1·46)	82 (14·6%)	2·2 (1·75-2·77)
No	11,209	4,187 (37·4%)	1	786 (7·0%)	1
**Bipolar disorder**					
Yes	340	100 (29·4%)	0·86 (0·71-1·05)	24 (7·1%)	1·23 (0·82-1·85)
No	11,432	4,334 (37·9%)	1	844 (7·4%)	1

**Depressive disorder**					
Yes	2,037	713 (35·0%)	1·01 (0·93-1·09)	138 (6·8%)	1·05 (0·87-1·26)
No	9,735	3,721 (38·2%)	1	730 (7·5%)	1
**Anxiety disorder**					
Yes	1,869	745 (39·9%)	1·15 (1·06-1·24)	178 (9·5%)	1·49 (1·26-1·75)
No	9,903	3,689 (37·3%)	1	690 (7·0%)	1
**Alcohol use disorder**					
Yes	2,961	1,169 (39·5%)	1·18 (1·10-1·26)	322 (10·9%)	2·01 (1·75-2·30)
No	8,811	3,265 (37·1%)	1	546 (6·2%)	1
**Drug use disorder**					
Yes	3,345	1,825 (54·6%)	2·39 (2·25-2·54)	336 (10·0%)	1·78 (1·55-2·04)
No	8,427	2,609 (31·0%)	1	532 (6·3%)	1
**Substance use disorder (alcohol or drug use disorder)**					
Yes	4,825	2,266 (47·0%)	1·88 (1·78-2·00)	479 (9·9%)	2·01 (1·76-2·30)
No	6,947	2,168 (31·2%)	1	389 (5·6%)	1
**Personality disorder**					
Yes	1,324	613 (46·3%)	1·5 (1·38-1·64)	184 (13·9%)	2·46 (2·09-2·90)
No	10,448	3,821 (36·6%)	1	684 (6·5%)	1
**Attention deficit hyperactivity disorder**					
Yes	608	210 (34·5%)	1·31 (1·14-1·50)	50 (8·2%)	1·82 (1·37-2·43)
No	11,164	4,224 (37·8%)	1	818 (7·3%)	1
**Other developmental or childhood disorder**					
Yes	777	352 (45·3%)	1·48 (1·33-1·65)	110 (14·2%)	2·57 (2·10-3·14)
No	10,995	4,082 (37·1%)	1	758 (6·9%)	1
**Prior self-harm**					
Yes	2,323	904 (38·9%)	1·16 (1·08-1·25)	218 (9·4%)	1·56 (1·34-1·82)
No	9,449	3,530 (37·4%)	1	650 (6·9%)	1

Collinearity estimates

Pairwise collinearity between baseline covariates measured with Cramer's V. No association is 0, full collinearity is 1.

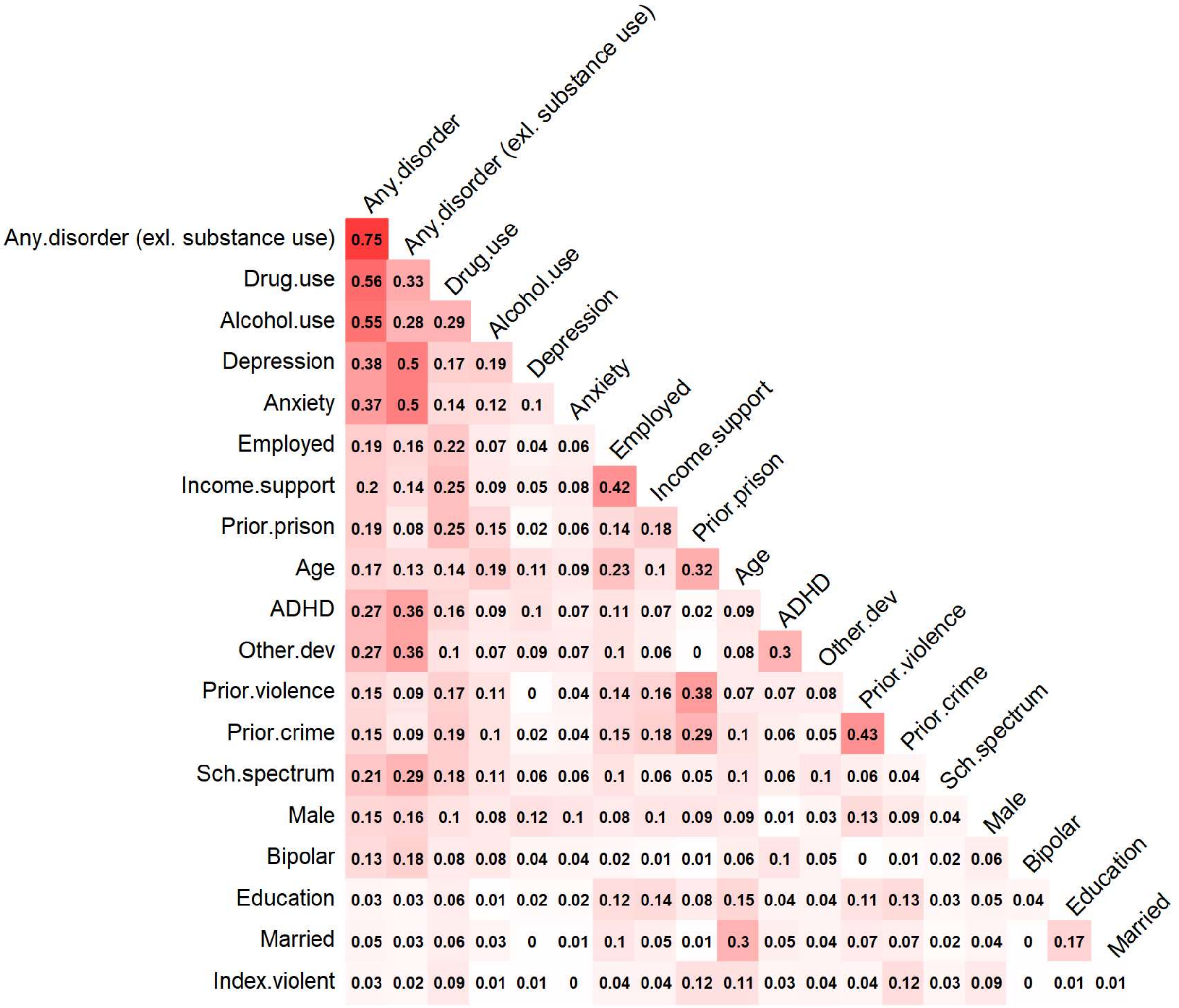

Same-sex full sibling cohort

Baseline characteristics and follow-up data of adult individuals with same-sex full siblings receiving community sentences from November 1, 1991, to December 31, 2013

	Men	Women	Total
Number of individuals	4,789 (94·3%)	292 (5·7%)	5,081 (100·0%)
**Baseline**			
Median age	24 (IQR: 20-31)	27·5 (IQR: 22-37)	24 (IQR: 20-32)
Married or in a registered partnership	305 (6·4%)	39 (13·4%)	344 (6·8%)
Employed	1,696 (35·4%)	60 (20·5%)	1,756 (34·6%)
Years of education			
< 9 yr	156 (3·3%)	33 (11·3%)	189 (3·7%)
9-11 yr	4,246 (88·7%)	239 (81·8%)	4,485 (88·3%)
≥ 12 yr	106 (2·2%)	8 (2·7%)	114 (2·2%)
Recipient of income support	2,207 (46·1%)	182 (62·3%)	2,389 (47·0%)
Prior criminal history	4,046 (84·5%)	237 (81·2%)	4,283 (84·3%)
Prior violent crime	2,207 (46·1%)	182 (62·3%)	2,389 (47·0%)
Prior imprisonment	1236 (25·8%)	61 (20·9%)	1297 (25·5%)
Index violent offence	2,292 (47·9%)	96 (32·9%)	2,388 (47·0%)
Any psychiatric disorder	1,865 (38·9%)	181 (62·0%)	2,046 (40·3%)
Any psychiatric disorder (excluding substance use)	1,170 (24·4%)	133 (45·5%)	1,303 (25·6%)
Schizophrenia spectrum disorder	120 (2·5%)	7 (2·4%)	127 (2·5%)
Bipolar disorder	39 (0·8%)	6 (2·1%)	45 (0·9%)
Depression	306 (6·4%)	39 (13·4%)	345 (6·8%)
Anxiety disorder	374 (7·8%)	56 (19·2%)	430 (8·5%)
Personality disorder	187 (3·9%)	31 (10·6%)	218 (4·3%)
Attention-deficit hyperactivity disorder	245 (5·1%)	12 (4·1%)	257 (5·1%)
Other developmental or childhood disorder	232 (4·8%)	21 (7·2%)	253 (5·0%)
Substance (drug or alcohol) use disorder	1,324 (27·6%)	120 (41·1%)	1,444 (28·4%)
Alcohol use disorder	739 (15·4%)	65 (22·3%)	804 (15·8%)
Drug use disorder	934 (19·5%)	97 (33·2%)	1,031 (20·3%)
**Follow-up data. General reoffending**			
Number of persons-year at risk	11,309·1	712·6	12,021·7
Incidents of reoffending during follow-up	2,902 (60·6%)	158 (54·1%)	3,060 (60·2%)
Median time to any new offence (months)			
All individuals	15·8 (IQR: 4·9-42·8)	18·6 (IQR: 5·9-44·9)	15·9 (IQR: 4·9-42·9)
Individuals with psychiatric disorder	12·0 (IQR: 3·6-29·7)	14·7 (IQR: 4·6-40·6)	12·3 (IQR: 3·7-30·4)
Individual without psychiatric disorder	20·2 (IQR: 6·1-50·3)	25·2 (IQR: 9·0-51·4)	20·2 (IQR: 6·2-50·4)
Reoffending rate (cumulative)			
1-year	1,690 (35·3%)	80 (27·4%)	1,770 (34·8%)
2-year	2,240 (46·8%)	114 (39·0%)	2,354 (46·3%)
3-year	2,524 (52·7%)	129 (44·2%)	2,653 (52·2%)
4-year	2,670 (55·8%)	141 (48·3%)	2,811 (55·3%)
5-year	2,749 (57·4%)	151 (51·7%)	2,900 (57·1%)
Died during follow-up	52 (1·1%)	1 (0·3%)	53 (1·0%)
Emigrated during follow-up	27 (0·6%)	0 (0%)	27 (0·5%)
**Follow-up data. Violent reoffending**			

Number of persons-year at risk	17,213·0	1,210·3	18,423·3
Incidents of reoffending during follow-up	966 (20·2%)	26 (8·9%)	992 (19·5%)
Median time to a violent offence (months)			
All individuals	32·6 (IQR: 11·9-66·9)	40·3 (IQR: 15·5-74·6)	32·9 (IQR: 12·4-67·4)
Individuals with psychiatric disorder	25·5 (IQR: 9·6-56·1)	39·8 (IQR: 14·6-73·1)	26·2 (IQR: 9·9-57·0)
Individual without psychiatric disorder	37·5 (IQR: 14·2-71·3)	42·0 (IQR: 16·2-77·1)	37·7 (IQR: 14·3-71·3)
Reoffending rate (cumulative)			
1-year	395 (8·2%)	7 (2·4%)	402 (7·9%)
2-year	594 (12·4%)	9 (3·1%)	603 (11·9%)
3-year	723 (15·1%)	14 (4·8%)	737 (14·5%)
4-year	812 (17·0%)	18 (6·2%)	830 (16·3%)
5-year	858 (17·9%)	19 (6·5%)	877 (17·3%)
Imprisoned during follow-up	936 (19·5%)	50 (17·1%)	986 (19·4%)
Died during follow-up	117 (2·4%)	10 (3·4%)	127 (2·5%)
Emigrated during follow-up	48 (1·0%)	2 (0·7%)	50 (1·0%)

Kaplan-Meier curves for observed general reoffending in individuals given community sentences stratified by sex and psychiatric disorder status

(A) Men

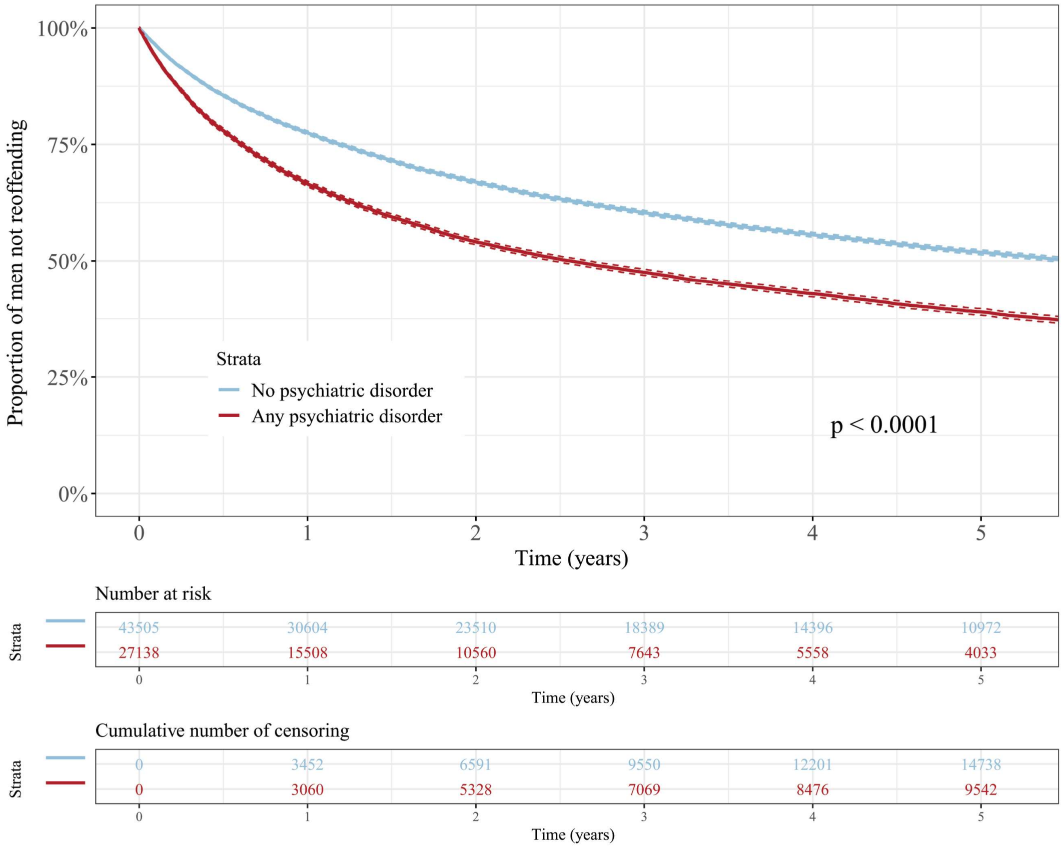

(B) Women

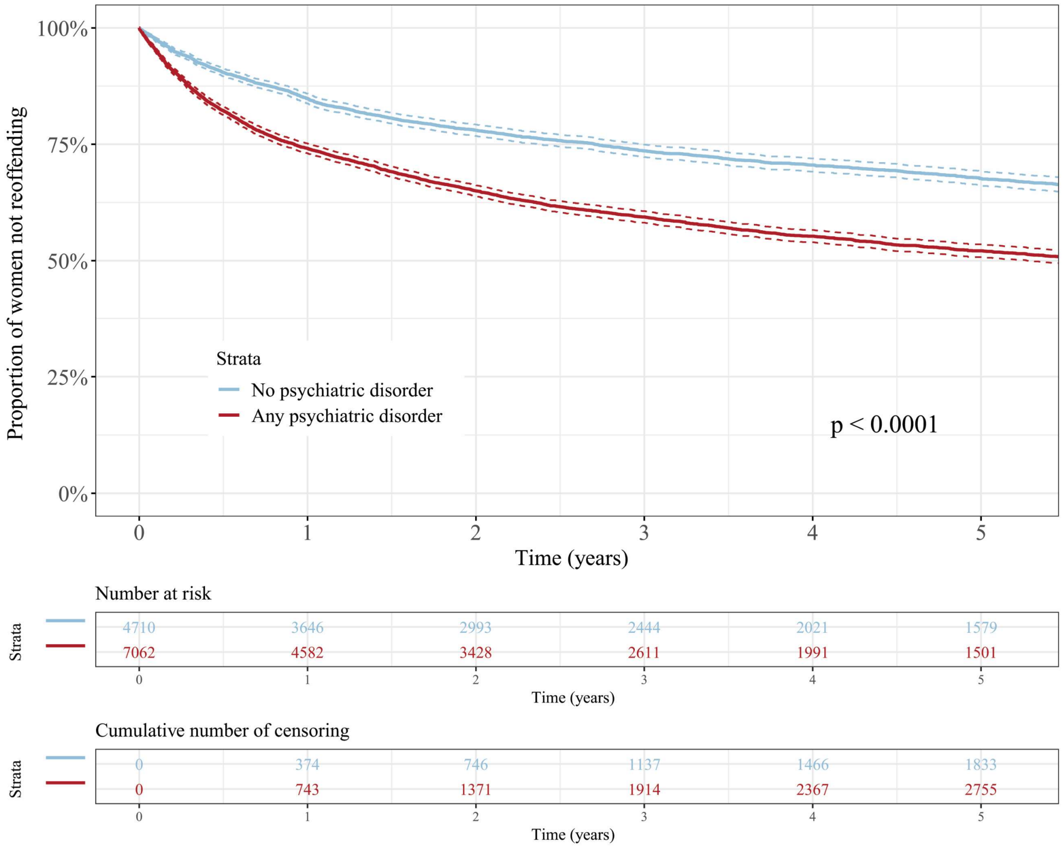

Association between individual psychiatric diagnoses and general reoffending in individuals given community sentences stratified by sex

Outcome: General reoffending

	Full cohort		Comparison between same-sex full siblings	
	N with diagnosis	Hazard ratio (95% CI)	Cases / disc. siblings	Hazard ratio (95% CI)
**Men (N = 70,643)**				
Any psychiatric diagnosis	27,138	1·59 (1·56-1·63)	978 / 1011	1·59 (1·35-1·87)
Any psychiatric diagnosis (excl. substance use)	18,047	1·27 (1·24-1·31)	782 / 815	1·30 (1·08-1·56)
Schizophrenia spectrum	2,032	1·37 (1·28-1·45)	112 / 127	1·67 (1·05-2·67)
Bipolar	690	1·02 (0·91-1·15)	39 / 43	1·58 (0·61-4·11)
Depression	5,447	1·04 (0·99-1·08)	278 / 300	0·88 (0·65-1·20)
Anxiety	5,604	1·21 (1·16-1·26)	327 / 353	1·34 (1·02-1·76)
Personality disorder	2,671	1·76 (1·67-1·85)	173 / 190	1·34 (0·92-1·95)
Attention-deficit hyperactivity	3,370	1·32 (1·26-1·39)	202 / 212	1·01 (0·71-1·44)
Other developmental or childhood	3,246	1·27 (1·21-1·34)	192 / 200	0·81 (0·58-1·14)
Substance (drug or alcohol) use disorder	18,680	1·90 (1·86-1·95)	788 / 821	1·66 (1·39-1·98)
Alcohol use disorder	11,569	1·50 (1·46-1·54)	554 / 580	1·33 (1·09-1·64)
Drug use disorder	11,864	2·27 (2·21-2·33)	592 / 626	2·00 (1·63-2·46)
**Women (N = 11,772)**				
Any psychiatric diagnosis	7,062	1·71 (1·61-1·82)	48 / 48	2·00 (0·87-4·59)
Any psychiatric diagnosis (excl. substance use)	5,486	1·27 (1·20-1·35)	65 / 66	1·53 (0·82-2·86)
Schizophrenia spectrum	563	1·32 (1·16-1·50)	7 / 7	..
Bipolar	340	0·87 (0·72-1·06)	6 / 7	..
Depression	2,037	1·02 (0·94-1·10)	35 / 37	0·96 (0·39-2·38)
Anxiety	1,869	1·15 (1·06-1·25)	44 / 46	1·41 (0·64-3·08)
Personality disorder	1,324	1·52 (1·40-1·66)	25 / 26	1·39 (0·56-3·50)
Attention-deficit hyperactivity	608	1·29 (1·12-1·48)	8 / 9	..
Other developmental or childhood	777	1·46 (1·31-1·63)	19 / 19	2·14 (0·52-8·73)
Substance (drug or alcohol) use disorder	4,825	1·91 (1·80-2·03)	53 / 56	1·89 (0·90-3·97)
Alcohol use disorder	2,961	1·20 (1·12-1·28)	41 / 40	0·67 (0·30-1·50)
Drug use disorder	3,345	2·40 (2·26-2·55)	46 / 49	2·50 (1·12-5·60)

Note: Risk factors and covariates were recorded at baseline (start of a sentence). Reported hazard ratios were adjusted for age. The estimates were not reported, if number of discordant pairs was less than 10 or the model returned infinite confidence intervals. Cases – the number of siblings-probands with a given diagnosis. Disc. siblings – siblings, discordant by a given diagnosis with their proband.

Population attributable fraction (PAF) estimations

PAFs for the outcomes during the first 2 years of the follow-up period.

Population attributable fraction (% [95% CI]) estimated from Cox regression models adjusted for age

	Any psychiatric disorder (including substance use)	Any psychiatric disorder (excluding substance use)	Substance use
**General reoffending**			
Men	13·6 (12·9-14·2)	4·8 (4·3-5·3)	13·3 (12·8-13·8)
Women	25·4 (22·6-28·2)	9·3 (7·0-11·6)	22·4 (20·3-24·5)
**Violent reoffending**			
Men	16·7 (15·3-18·1)	9·5 (8·3-10·6)	13·6 (12·4-14·8)
Women	40·3 (33·8-46·9)	28·8 (23·1-34·5)	29·7 (24·4-35·0)

Population attributable fraction (% [95% CI]) estimated from Cox regression models adjusted for age, criminal history, and sociodemographic factors

	Any psychiatric disorder (including substance use)	Any psychiatric disorder (excluding substance use)	Substance use
**General reoffending**			
Men	5·5 (4·8-6·3)	0·7 (0·2-1·2)	6·7 (6·2-7·3)
Women	15·7 (12·5-18·9)	4·8 (2·4-7·2)	14·4 (12·0-16·7)
**Violent reoffending**			
Men	8·3 (6·6-10·0)	4·4 (3·1-5·6)	7·3 (5·9-8·7)
Women	30·9 (22·7-39·0)	20·5 (13·7-27·2)	24·2 (18-30·4)

The association between a number of separate psychiatric diagnoses an individual has and reoffending

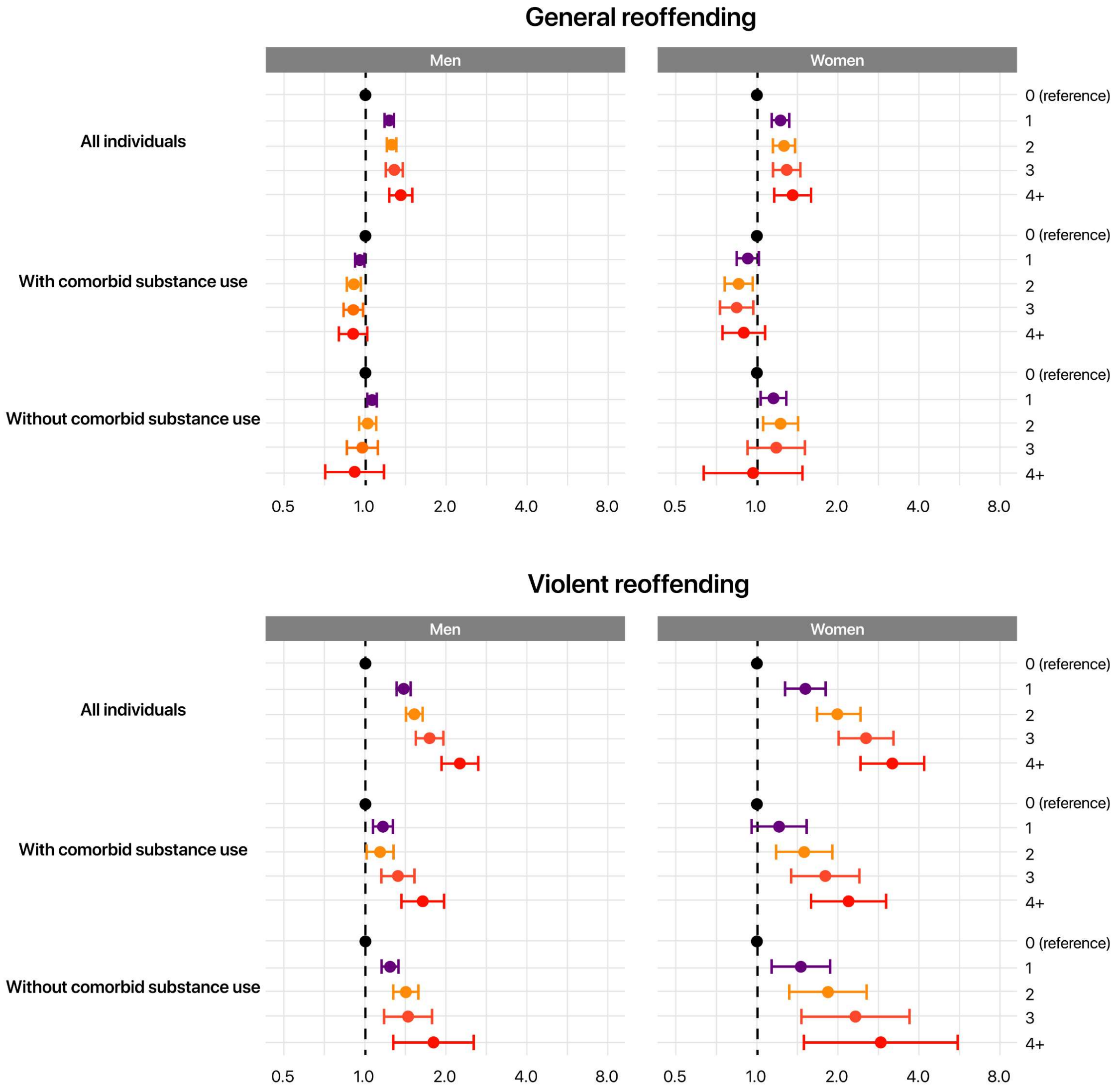

Kaplan-Meier curves for observed violent reoffending in individuals given community sentences stratified by sex and psychiatric disorder

(A) Men

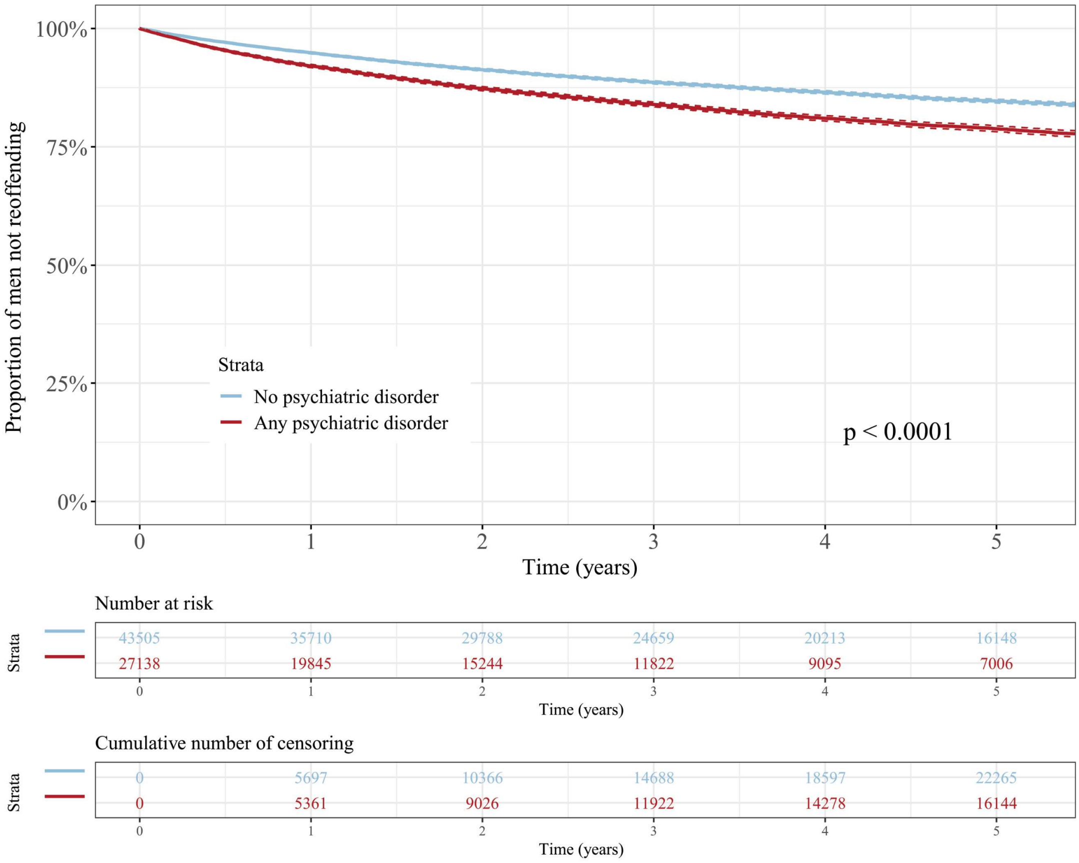

(B) Women

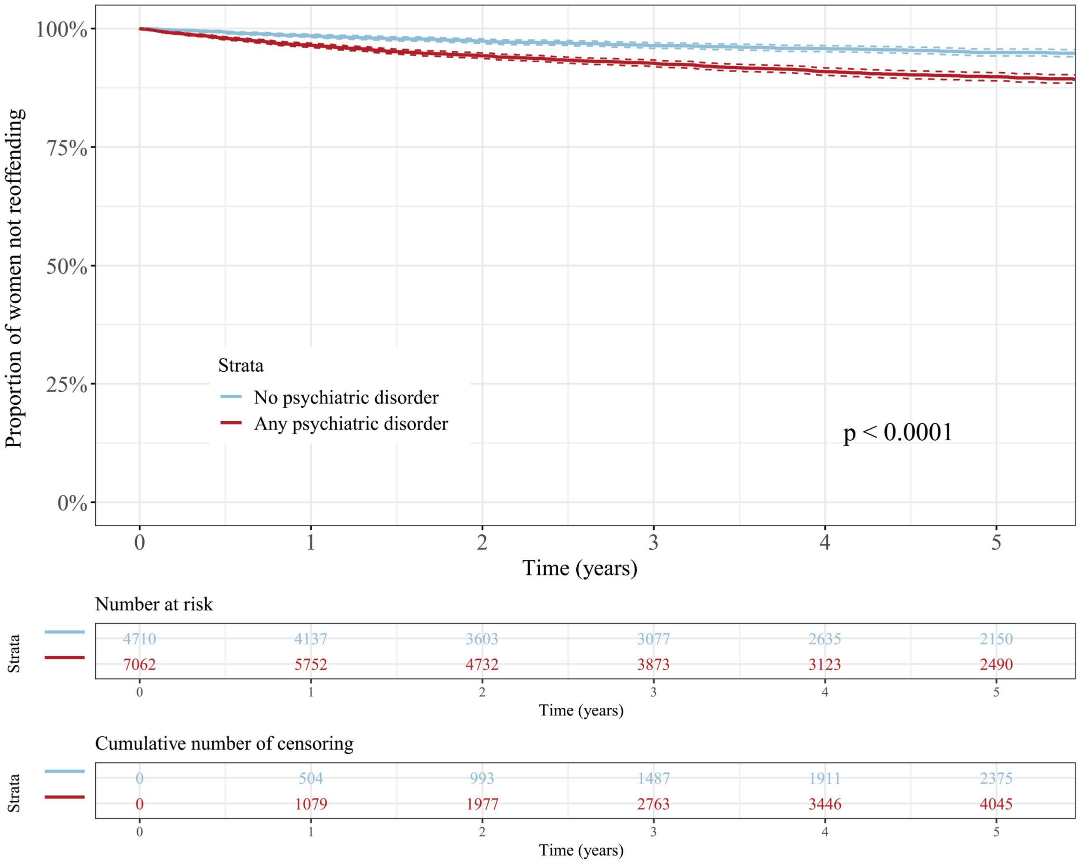

Association between individual psychiatric diagnoses and violent reoffending in individuals given community sentences stratified by sex

Outcome: Violent reoffending

	Full cohort		Comparison between same-sex full siblings	
	N with diagnosis	Hazard ratio (95% CI)	Cases / disc. siblings	Hazard ratio (95% CI)
**Men (N = 70,643)**				
Any psychiatric diagnosis	27,138	1·60 (1·54-1·66)	978 / 1011	1·53 (1·16-2·01)
Any psychiatric diagnosis (excl. substance use)	18,047	1·47 (1·41-1·53)	782 / 815	1·44 (1·05-1·96)
Schizophrenia spectrum	2,032	1·84 (1·67-2·02)	112 / 127	3·70 (1·37-10·02)
Bipolar	690	1·13 (0·91-1·40)	39 / 43	1·15 (0·22-6·04)
Depression	5,447	1·10 (1·02-1·19)	278 / 300	0·92 (0·55-1·54)
Anxiety	5,604	1·28 (1·20-1·38)	327 / 353	1·52 (0·91-2·54)
Personality disorder	2,671	2·18 (2·00-2·37)	173 / 190	2·03 (1·00-4·09)
Attention-deficit hyperactivity	3,370	1·57 (1·44-1·71)	202 / 212	1·26 (0·72-2·20)
Other developmental or childhood	3,246	1·68 (1·56-1·82)	192 / 200	1·20 (0·70-2·04)
Substance (drug or alcohol) use disorder	18,680	1·71 (1·64-1·78)	788 / 821	1·68 (1·24-2·27)
Alcohol use disorder	11,569	1·72 (1·63-1·80)	554 / 580	1·61 (1·13-2·28)
Drug use disorder	11,864	1·73 (1·65-1·81)	592 / 626	1·61 (1·13-2·30)
**Women (N = 11,772)**				
Any psychiatric diagnosis	7,062	2·19 (1·88-2·54)	48 / 48	0·23 (0·02-2·18)
Any psychiatric diagnosis (excl. substance use)	5,486	1·92 (1·68-2·20)	65 / 66	1·35 (0·30-6·10)
Schizophrenia spectrum	563	2·44 (1·94-3·08)	7 / 7	..
Bipolar	340	1·30 (0·86-1·95)	6 / 7	..
Depression	2,037	1·09 (0·91-1·31)	35 / 37	..
Anxiety	1,869	1·52 (1·29-1·79)	44 / 46	0·25 (0·03-2·25)
Personality disorder	1,324	2·61 (2·21-3·07)	25 / 26	3·58 (0·36-35·72)
Attention-deficit hyperactivity	608	1·71 (1·28-2·28)	8 / 9	..
Other developmental or childhood	777	2·43 (1·99-2·97)	19 / 19	..
Substance (drug or alcohol) use disorder	4,825	2·09 (1·83-2·39)	53 / 56	0·54 (0·13-2·20)
Alcohol use disorder	2,961	2·16 (1·88-2·49)	41 / 40	0·91 (0·17-4·87)
Drug use disorder	3,345	1·81 (1·58-2·08)	46 / 49	0·22 (0·02-2·73)

Note: Risk factors and covariates were recorded at baseline (start of a sentence). Reported hazard ratios were adjusted for age. The estimates were not reported, if number of discordant pairs was less than 10 or the model returned infinite confidence intervals. Cases – the number of siblings-probands with a given diagnosis. Disc. siblings – siblings, discordant by a given diagnosis with their proband.

References

1 Swedish Prison and Probation Service. Probation. 2021. https://www.kriminalvarden.se/swedish-prison-and-probation-service/probation/ (accessed Aug 7, 2022).

2 Bosly S, Flore D, Honhon A, Maggio J, editors. Probation measures and alternative sanctions in the European Union. Intersentia Publishing Limited; 2012.

3 Murley C, Friberg E, Hillert J, Alexanderson K, Yang F. Validation of multiple sclerosis diagnoses in the Swedish National Patient Register. *Eur J Epidemiol* 2019; 34(12): 1161-9.

4 Swedish National Council for Crime Prevention. All conviction decisions, by principal offence and year. 2022. https://bra.se/bra-in-english/home/crime-and-statistics/crime-statistics.html (accessed Nov 7, 2022).
